# Exploration of nonlinear optical properties of 4-methyl-4H-1,2,4-triazol-3-yl)thio)-*N*-phenylpropanamide based derivatives: experimental and DFT approach

**DOI:** 10.1038/s41598-024-51788-z

**Published:** 2024-02-01

**Authors:** Muhammad Irfan, Hammad Ali Khan, Shamsa Bibi, Gang Wu, Akbar Ali, Samreen Gul Khan, Norah Alhokbany, Faiz Rasool, Ke Chen

**Affiliations:** 1 Department of Chemistry, Faculty of Physical Science, Government College University, Faisalabad, 38000 Pakistan; 2 Department of Chemistry, University of Agriculture, Faisalabad, Pakistan; 3 Department of Infectious Diseases, The Affiliated Hospital of Southwest Medical University, Luzhou, 646000 China; 4 Department of Chemistry, College of Science, King Saud University, 11451 Riyadh, Saudi Arabia; 5 Institute of Chemical Sciences, Bahauddin Zakariya University, Multan, 60800 Pakistan

**Keywords:** Chemistry, Optics and photonics

## Abstract

Triazoles, nitrogen-containing heterocycles, have gained attention for their applications in medicinal chemistry, drug discovery, agrochemicals, and material sciences. In the current study, we synthesized novel derivatives of N-substituted 2-((5-(3-bromophenyl)-4-methyl-4H-1,2,4-triazol-3-yl)thio)-N-phenylpropanamide and conducted a comprehensive investigation using density functional theory (DFT). These novel structural hybrids of 1,2,4-triazole were synthesized through the multi-step chemical modifications of 3-bromobenzoic acid **(1)**. Initially, compound 1 was converted into its methyl-3-bromobenzoate **(2)** which was then transformed into 3-bromobenzohydrazide **(3)**. The final step involved the cyclization of compound 3, producing its 1,2,4-triazole derivative **(4)**. This intermediate was then coupled with different electrophiles, resulting in the formation of the final derivatives **(7a–7c)**. Additionally, the characterization of these triazole-based compounds (**7a, 7b**, and **7c**) were carried out using techniques such as IR, HNMR, and UV–visible spectroscopy to understand their structural and spectroscopic properties. The DFT study utilized M06/6-311G(d,p) functional to investigate geometrical parameters, HOMO–LUMO energies, natural bond orbital analyses, transition density matrix (TDM), density of states, and nonlinear optical (NLO) properties. The FMO analysis revealed that compound **7c** exhibited the lowest band gap value (4.618 eV). Notably, compound **7c** exhibited significant linear polarizability (4.195 >  × 10^–23^) and first and second hyperpolarizabilities (6.317 >  × 10^–30^, 4.314 × 10^–35^), signifying its potential for nonlinear optical applications. These NLO characteristics imply that each of our compounds, especially **7c**, plays a crucial part in fabricating materials showing promising NLO properties for optoelectronic applications.

## Introduction

Compounds containing heterocyclic rings are important in the development of new classes of medicinal compounds^1,2^. The broad biological and pharmacological spectrum of heterocyclic molecules has sparked the interest of researchers in recent years. Particularly, heterocyclic compounds containing azoles are ranked as of special interest due to their strong synthetic and pharmaceutical applications^3,4^. Heterocyclic compounds have been shown to exhibit great selectivity of action, minimal toxicity, and a therapeutic impact comparable to that of traditional medications^5^. Heterocyclic compounds are specified to have carbon, oxygen, and other elements like nitrogen and sulfur within their cyclic rings^6^. Nitrogenous containing heterocycles such as triazoles and their derivatives are interesting as they exhibit many structural characteristics of bioactive compounds^7,8^. In 2023, Khram et al. reported the development of a novel method for the synthesis of 1,2,4-triazole- and tetrazole-containing thiopyrano[2,3-b]quinolines and highlighted the potential of these compounds as antiviral drugs^9^. In the same year, Maghraby et al. conducted a comprehensive study to investigate a novel series of 1,2,3-triazole/1,2,4-triazole hybrids as antiproliferative agents targeting aromatase enzymes. Their findings highlighted their potential as therapeutic agents. ^10^ In many research studies, 1,2,4-triazoles have been shown to possess powerful biological properties, including antibacterial^11,12^, antimicrobial^13,14^, antifungal^15^, anticancer^16,17^, antimycotic activity^18^, antinociceptive^19^, antioxidant^20^, anticonvulsant^21,22^, antiviral^23^, anti-inflammatory^24^, and analgesic properties^25^.

Triazole building blocks are utilized in the synthesis of drugs^26^, agrochemicals^27^, dyes^28^, and pharmaceuticals^29,30^. They also find application as corrosion inhibitors^31^, stabilizers in polymers^32^, and ligands in coordination chemistry^33^. Moreover, triazoles are utilized in the development of materials with unique properties, such as conducting polymers and sensors^34,35^.

Additionally, these compounds hold promise for nonlinear optical (NLO) applications in various scientific fields, including biophysics, chemical dynamics, surface interface presentations, medicine, materials, and nuclear sciences^36,37^. Computational calculations employing DFT are used to predict the electronic properties, including non-covalent interactions, stability, magnetism, and nonlinear optical behavior, of organic compounds^38,39^. In this context, DFT analysis has been employed to investigate the electronic properties, specifically the nonlinear optical behavior, of 1,2,4-triazole derivatives.

Islam et al., in 2023, conducted a comprehensive study to investigate the influence of strain on the optical properties of APbCl_3_ (A = K, Rb, Cs) perovskites using density-functional theory (DFT) models. The researchers found that strain can improve the optical performance of these perovskites, resulting in enhanced absorption and reduced optical losses^40^. In a separate study conducted by Mushtaq et al. in 2023, the theoretical adsorption of NOx gas molecules on the surface of CoFeMnSi quaternary Heusler alloys was investigated using density functional theory calculations. The results of the study showed that NOx molecules act as charge acceptors by attracting charge from the surface atoms of the Heusler alloys. This charge transfer process enhances the surface reactivity and could potentially lead to improved gas sensing capabilities^41^.

Rahman et al. (2023) conducted a comprehensive investigation on the optical and electronic properties of Sr_3_NCl_3_, with the aim of gaining valuable insights into its potential applications in the field of optoelectronics and solar cell design. Their findings highlighted the potential of Sr_3_NCl_3_ to exhibit strong visible light absorption properties, with potential variations observed under increasing compressive (tensile) strain^42^. In another study, Rahman et al. (2023) conducted a detailed analysis of the optical properties of Ca_3_AsI_3_ using first-principles density-functional theory. Their research revealed that Ca_3_AsI_3_ exhibits exceptional optical properties, making it highly suitable for efficient visible light absorption^43^. Furthermore, Rahman et al. (2023) utilized the first principles DFT to explore how the optical and electronic characteristics of Ca_3_PI_3_ are impacted by strain. Their findings demonstrated the strong absorption capabilities of Ca_3_PI_3_ in the visible range^44^. Overall, these studies by Rahman et al. provide valuable insights into the optical and electronic properties of Sr_3_NCl_3_, Ca_3_AsI_3_, and Ca_3_PI_3_, highlighting their potential in optoelectronics and solar cell design.

Joe et al. (2023) found that benzodiazepine compounds with NO_2_ and Br substitutions exhibit 2-order hyperpolarizability as well as self-focusing switching. Results indicate their applications in optoelectronics and photonics^45^. Khalid et al. (2023) formulated chromophores that exhibited significant nonlinear optical (NLO) results. Their findings reveal that the compound PCMD8 demonstrated the smallest band gap of 2.048 eV, efficient intramolecular charge transfer, and maximum energy of stabilization were investigated^46^. Zainuri et al. (2023) investigated compounds that demonstrate promising potential in nonlinear optical (NLO) applications, such as optical limiting devices, due to their excellent structural stability and strong intermolecular interaction^47^.

This study aims to achieve two main objectives:1. Synthesis of novel 1,2,4-triazole core embedded density functionalized organic building blocks. 2. Exploration of optoelectronic behavior using density functional theory^48,49^. According to a literature review, NLO characteristics of triazole-based materials have not been published yet. To fill this knowledge gap, the current work uses systematic structure–property interactions to design and produce triazole-based isomers for energetic materials. The three triazole based derivatives; 2-((5-(3-bromophenyl)-4-methyl-4H-1,2,4-triazol-3-yl)thio)-N-(4-chloro-3-(trifluoromethyl)phenyl)propanamide, 2-((5-(3-bromophenyl)-4-methyl-4H-1,2,4-triazol-3-yl)thio)-N-(2-chlorophenyl)propanamide, and 2-((5-(3-bromophenyl)-4-methyl-4H-1,2,4-triazol-3-yl)thio)-N-(3-nitrophenyl)propenamide were synthesized. The present study is of vital importance for evaluating the NLO uses of organic molecules. Hopefully, this study will serve as the basis for developing entirely novel triazole-based organic dyes with exceptional NLO properties.

## Materials and methods

### General

Standard chemicals from Sigma Aldrich, Acros Chemicals, Macklin, and TCI were used without any additional purification. Silica gel 60 254F plates (Merck Germany) was used to perform TLC for reaction progress. UV–visible spectra were measured and analyzed with high accuracy and precision by the Hitachi UV–Visible Double Beam Spectrophotometer. A Stuart melting point apparatus was used to determine the melting point of the samples.

### Chemistry

**Figure 1:**
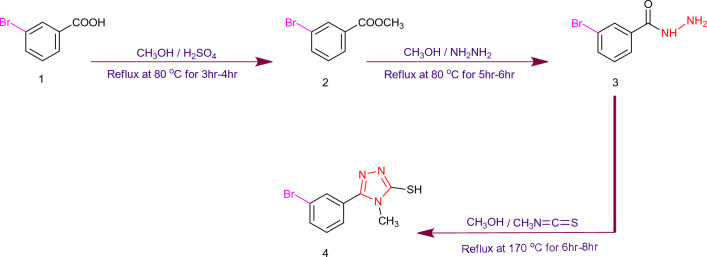
Synthesis of 1,2,4-Triazole.

**Figure 2:**
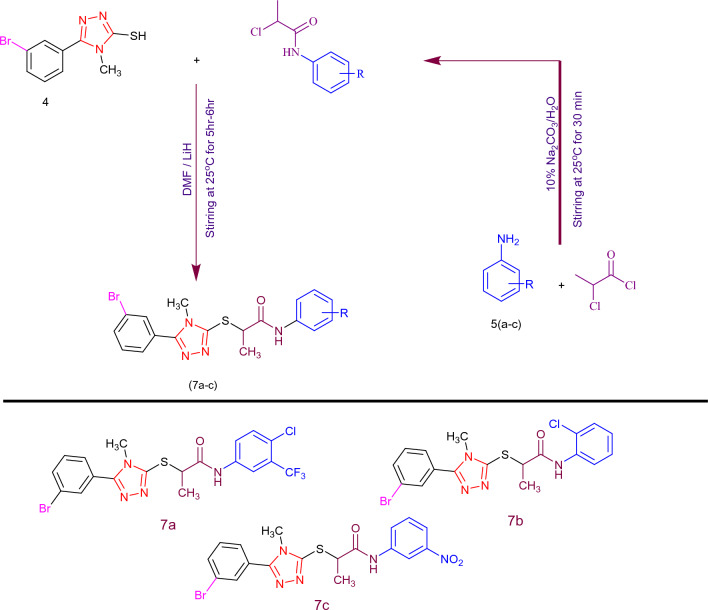
Synthesis of *N*-substituted triazole based derivatives.

A multi-step (three-step) procedure was adopted for the synthesis of triazoles starting form 3-bromobenzoic acid as precursor. The synthetic route follows the synthesis of methyl-3-bromobenzoate (2) from 3-bromobenzoic acid (1) fallowed by converting it to 3-bromobenzohydrazide (3) and then converting that hydrazide into 5-(3-bromophenyl)-4-methyl-4H-1,2,4-triazole-3-thiol (4) as shown in Fig. 1. On the other hand, 2-chloropropanoyl chloride was reacted with different anilines (5a–5c) to obtain the desired N-substituted-2-Chloro-N-phenylpropanamide (6a–6c). Finally, the derivatives (7a–7c) were prepared by the reaction between (4) and (6a-6c) under stirring with DMF as a solvent and LiH as a catalyst, as shown in Fig. 2. Complete structures of (7a–7c) have been shown in Fig. S22.

### General Synthesis Protocol for 2-((5-(3-bromophenyl)-4-methyl-4H-1,2,4-triazol-3-yl)thio)-*N-*phenylpropanamide(7a–7c)

The triazole embedded derivatives were synthesized by 24 h of *N-*substituted 2-chloro-*N-*phenylpropanamide (0.1 g) (5a–c), 5-(3-bromophenyl)-4-methyl-4H-1,2,4-triazole-3-thiol (0.1 g), LiH (0.05 g) and DMF (4 ml) in a round-bottom flask. TLC was used to check reaction progress. After reaction completion, the achieved precipitates were filtered off, washed with distilled water, and dried.

#### 2-((5-(3-bromophenyl)-4-methyl-4H-1,2,4-triazol-3-yl)thio)-N-(4-chloro-3-(trifluoromethyl)phenyl)propanamide (7a)

Light blue amorphous. Percentage yield: 88%. MP: 182–184 °C. (λ _max_ = 298 nm).IR (KBr, cm^−1^) 3220 (NH), 1695 (C=O), 1541 (N=N), 1483 (Ar–C=C), 1131 (C–N), 1032 (C–F), 728 (C–Cl). ^1^H-NMR (*δ*/ppm, 400 MHz, CDCl_3_): 10.87 (H-NH s, 1H), 8.15 (s, 1H, H-2′′), 7.79 (s, H-2′, 1H), 7.7 (H-6′,d, 1H, *J* = 12 Hz), 7.67 (1H, H-5′,t, *J* = 12 Hz), 7.56 (H-4′,d, *J* = 8 Hz, 1H), 7.44 (H-5′′,d, 1H, *J* = 8 Hz), 7.4 (H-6′′,d, 1H,* J* = 8 Hz), 4.58 (H–S-CH, q, 1H, *J* = 8 Hz), 3.61 (CH_3_–N, s, 3H), 1.71 (CH_3_–CH, d, 3H, *J* = 4 Hz). ^13^C NMR (*δ*/ppm, 101 MHz, CDCl3): 169.57, 154.83, 152.57, 137.33, 133.82, 131.70, 131.49, 128.74, 128.43, 128.09, 127.72, 127.01, 126.49, 123.68, 123.18, 118.84, 44.86, 31.98, 16.77. GCMS: 519. Elemental Analysis: C, 43.91; H, 2.91; N, 10.78.

#### 2-((5-(3-bromophenyl)-4-methyl-4H-1,2,4-triazol-3-yl)thio)-N-(2-chlorophenyl) propanamide (7b)

White amorphous. Percentage yield: 82%. MP : 118–120 °C. (λ _max_ = 297 nm). IR (KBr, cm^−1^) 3272 (NH), 1677 (C=O), 1591 (N=N), 1440 (Ar–C=C), 1163 (C–N), 747 (C–Cl). ^1^H-NMR (*δ*/ppm, 400 MHz, CDCl_3_): 9.73 (s, 1H, H-NH), 8.34 (H-6′,d, 1H, *J* = 8 Hz), 7.77 (H-2′, s, 1H), 7.66 (H-3′′,d, *J* = 8 Hz, 1H), 7.55 (H-4′,d, 1H, *J* = 8 Hz), 7.41 (H-5′,t,* J* = 8 Hz, 1H), 7.37 (H-5′′,t, *J* = 8 Hz, 1H,), 7.24 (H-6′′, d, 1H, *J* = 8 Hz), 7.05 (t, H-4′′, 1H, *J* = 8 Hz), 4.82 (H–S-CH, q, *J* = 8 Hz, 1H), 3.58 (CH_3_–N, s, 3H), 1.74 (CH_3_–CH, d, *J* = 8 Hz, 3H,). ^13^C NMR (*δ*/ppm, 101 MHz, CDCl3): 169.46, 154.64, 152.26, 134.84, 133.63, 131.57, 130.55, 129.32, 127.93, 127.29, 127.21, 125.09, 124.38, 123.06, 122.54, 44.35, 31.87, 16.84. GCMS: 451. Elemental Analysis: C, 47.85; H, 3.57; N, 12.40.

#### 2-((5-(3-bromophenyl)-4-methyl-4H-1,2,4-triazol-3-yl)thio)-N-(3-nitrophenyl)propanamide (7c)

Yellow amorphous. Percentage yield: 93%. MP: 180–182 °C. (λ _max_ = 296 nm). IR (KBr, cm^−1^) 3226 (NH), 1733 (C=O), 1694 (N=C), 1558 (N=N), 1522 (NO_2_), 1464 (Ar–C=C), 1164 (C–N). ^1^H-NMR (*δ*/ppm, 400 MHz, CDCl_3_): 11.0 (H-NH, s, 1H), 8.7 (H-2′′, s, 1H), 7.93 (H-6′,d, *J* = 8 Hz, 1H), 7.83 (H-4′′,d, *J* = 8 Hz, 1H), 7.79 (H-2′, s, 1H), 7.69 (H-6′′,d, 1H, *J* = 8 Hz,), 7.58 (H-4′,d, 1H, *J* = 8 Hz), 7.45 (H-5′′, t, 1H, *J* = 4 Hz), 7.42 (t, 1H, *J* = 4 Hz, H-5′), 4.61 (H–S–CH, q, *J* = 8 Hz, 1H,), 3.63 (CH_3_–N, s, 3H), 1.73 (CH_3_–CH, d, 3H, *J* = 8 Hz). ^13^C NMR (*δ*/ppm, 101 MHz, CDCl3): 169.61, 154.77, 152.74, 148.55, 139.51, 133.90, 131.53, 130.67, 129.49, 127.56, 127.12, 125.44, 123.19, 118.66, 114.58, 45.04, 32.13, 16.76. GCMS: 462. Elemental Analysis: C, 46.76; H, 3.49; N, 15.15.

## Results and discussion

The spectroscopic examination identified synthesis of the compounds, as illustrated in Figs. S1–S21. Following the determination of their structural conformation, a DFT investigation was conducted on the newly synthesized molecules featuring 1,2,4-triazole functionalities. The specifics of this exploration are discussed below.

### Frontier molecular orbitals (FMO)

To explore the optical as well as electronic properties of compounds, FMO analysis is a remarkable tool to evaluate the probability of intramolecular charge transfer (ICT)^50,51^. HOMO demonstrates the ability to donate an electron and is regarded as valence band, while LUMO reflects the capacity to accept an electron and is regarded as conduction band. FMOs are the substantial source of transition energies that occur when an electron excites from the HOMO to the LUMO. The energy difference (∆*E* = *E*_LUMO_–*E*_HOMO_) of compounds is directly associated with their kinetic stability and chemical reactivity. A reduced energy difference (∆*E*) value results in increased molecular polarizability, leading to an enhanced nonlinear optical (NLO) response^52^. *E*_LUMO_, *E*_HOMO,_ and ∆*E* values for **A**–**C** were calculated, and the outcomes are in Table 1.

**Table 1:** The *E*_HOMO_, *E*_LUMO,_ and $\Delta$ *E* of **A-C**, units in *eV.*

Compounds	*E*_*HOMO*_	*E*_*LUMO*_	Δ*E*
A	− 7.128	− 1.491	5.637
B	− 6.973	− 1.358	5.615
C	− 7.144	− 2.526	4.618

The findings from Table 1 illustrates that by varying different groups in fragment 3, band gap of synthesized compounds vary greatly. Compounds with large ∆*E* need more HOMO to LUMO energy of transition than those with less energy difference hence making them preferable for NLO devices^53–55^. Therefore, compounds with lower band gaps are compelling in the opto-electronic domain. In these synthesized compounds, fragments 1 and 2 are kept unaltered, while fragment 3 is modified, which results in different **Δ*E*** values. The HOMO energies of synthesized molecules 7**a, 7b,** and **7c** are determined as − 7.128, − 6.973, and − 7.144 eV, respectively, whereas the LUMO energies are calculated as − 1.491, − 1.358, and − 2.526 eV, respectively. Consequently, the DFT-based computed HOMO–LUMO energy gaps of synthesized molecules are 5.637, 5.615, and 4.618 eV, respectively.

Among the synthesized compounds, the maximum ∆*E* value (5.637 eV) is found in **A**, which has a chloro group at the para position, and CF_3_ group at the meta position. Due to negative inductive effect (−I) the chloro group only slightly withdraws electrons, and it also releases electrons via resonance. Here, the resonance effect dominates over the inductive effect and thus reduces the charge transference. Moreover, the CF_3_ group is an electron withdrawing group due to the presence of three fluoro groups on the same carbon atom. Subsequently **B** with two chloro groups in its structure showed reduced band gap. This chloro group at the ortho position has a stronger electron-withdrawing effect, it would increase the charge transference in the molecule and potentially reduce the band gap. Furthermore, the compound **C**, with a meta-positioned NO2 group, exhibits the lowest band gap value, ascribed to the strong electron-withdrawing nature of the nitrogen atom, resulting in improved charge transfer and a pronounced push–pull mechanism. Overall, the energy gap is observed in the following decreasing order: **A > B > C**. Additionally, the outcomes for HOMO-1, LUMO + 1, HOMO-2, and LUMO + 2 energies, as listed in Table S1, provides further insights into the electronic structure and exciton dynamics of these compounds.

**Figure 3:**
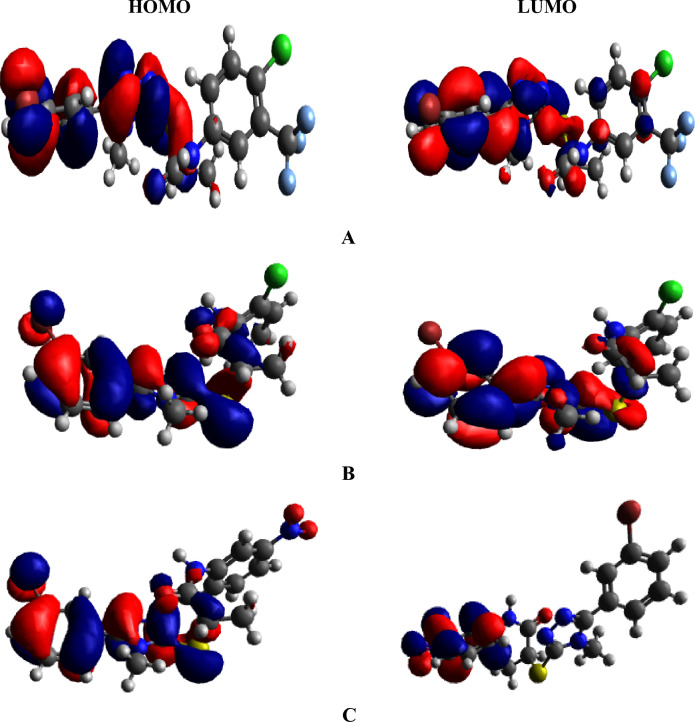
The HOMOs and LUMOs of the compounds (**A**)–(**C**), illustrate the electronic cloud over the orbitals.

Hence, among the three synthesized molecules (**A**, **B**, **C**) a smaller ∆*E* value is observed in **C**. The orbital diagrams of HOMO and LUMO are presented in Fig. 3. For HOMO, the charge density of synthesized compounds resides mainly on fragments 1 and 2, while LUMO charge density is hugely located on fragment 1 and partly on fragment 2. These orbital diagrams are useful for analysing effective charge transfer in the synthesized compounds. Therefore, high charge transition makes the synthesized compounds efficient materials for advanced NLO devices.

### Global reactivity parameters (GRPs)

Band gap is the most significant factor for the estimation of the global reactivity parameters (GRPs), such as electron affinity (*EA*)^56^, ionization potential (*IP*)^57^, global hardness (*η*)^58^, global softness (*σ*)^59^, chemical potential (*μ*)^60^, global electrophilicity index (*ω*)^61^, and electronegativity (*X*)^62^. It is an effective approach to predict global trends in chemical reactivity using these characteristics. The *EA* and *IP* amplitudes of chemical compounds are used for identifying their ability to capture and donate electrons^63^. The determination of electrophilic strength in chromophores relies on these parameters, where higher values of chemical potential (*μ*) and hardness (*η)* indicate increased kinetic stability. Additionally, these variables are directly linked to *∆E* values and exhibit an inverse relationship with global softness (*σ*). Therefore, molecules having smaller ∆*E* are more reactive, stable, and soft with high polarization and serve as a finer competitor in providing the best NLO response^64^. Table 2 shows the outcomes for the computed GRPs of the examined chromophores.

**Table 2:** Computed global reactivity parameters of **A-C,** units in *eV.*

Compounds	*IP*	*EA*	*X*	*Μ*	*Η*	*ω*	*σ*	*ΔNmax*
A	7.128	1.491	4.309	− 4.309	2.818	3.294	0.177	1.529
B	6.973	1.358	4.165	− 4.165	2.807	3.090	0.178	1.483
C	7.144	2.526	4.835	− 4.835	2.309	5.062	0.216	2.093

By using Koopmans′ theorem (Eqs. 1–7), the chemical hardness (*η*), chemical potential (*μ*), electronegativity (*X*), global softness (*σ*), and electrophilicity index (*ω*) are calculated^65,66^.

1 $$IP = \, - E_{HOMO}$$

2 $$EA = - E_{LUMO}$$

Electronegativity (*X*) and chemical potential (*μ*) are calculated using Koopmans’s theory as:

3 $$X=-\frac{\left[{E}_{{\text{LUMO}}}+{E}_{{\text{HOMO}}}\right]}{2}$$

4 $$\mu =\frac{{E}_{HOMO}{+E}_{LUMO}}{2}$$

The global softness (*σ*) and chemical hardness (*η*) can be calculated through Eqs. (5) and (6).

5 $$\eta =IP-{\text{EA}}$$

6 $$\sigma =\frac{1}{\eta }$$

The electrophilicity index (*ω*) is reported by Parr et al.^43,67^. as:

7 $$\omega =\frac{{\mu }^{2}}{2\eta }$$

From Table 2, compound **C** exhibits the highest ionization potential (*IP*) value among the synthesized chromophores (7.144 eV), suggesting efficient charge distribution among its fragments. In contrast, compound **B** exhibits the lowest ionization potential (*IP*) value of 6.973 eV, while compound **C** has the highest value of 7.144 eV. The ionization potential values follow the order **B < A < C**. Additionally, compound **C** demonstrates the highest electron affinity (*EA*) value of 2.526 eV, while compound **B** has the lowest value of 1.358 eV. It is noteworthy that the ionization potential values are generally higher than the electron affinity values for compounds **A, B**, and **C**. According to the literature, higher *IP* values indicate more chemical inertness and stability^68^. Chemical potential (*μ*) describes the molecular stability^69^ by relating to molecular electronegativity. The negative *μ* value indicate that the compound accepts electrons readily^70^. Similarly, the negative chemical potential values shown by the synthesized compounds indicate their stable nature.

The order of global hardness (*η*) follows the trend **C < B < A**, correlating with the increasing energy gap (*E*_*gap*_). This indicates that harder molecules with higher ∆*E* values exhibit greater stability and lower reactivity. Global softness (*σ*) is also linked to chemical potential and plays a role in understanding reactivity and stability. The increasing order of *σ* is given as **A** < **B** < **C**. This ascending order is opposite to the increasing energy gap order, representing **A** (0.177 eV) with the least reactivity; however, **C** (0.216 eV^−1^) is highly reactive showing highest softness value among the synthesized compounds. Overall, *E*_*gap*_ order and global reactivity descriptors have a very good association. Low-lying *E*_*gap*_ is admitted to showing high molecular nonlinear behavior. The remarkable nonlinear optical (NLO) responses observed in the tested systems strongly indicate their potential for significant applications in optoelectronics.

### Density of states (DOS) analysis

**Figure 4:**
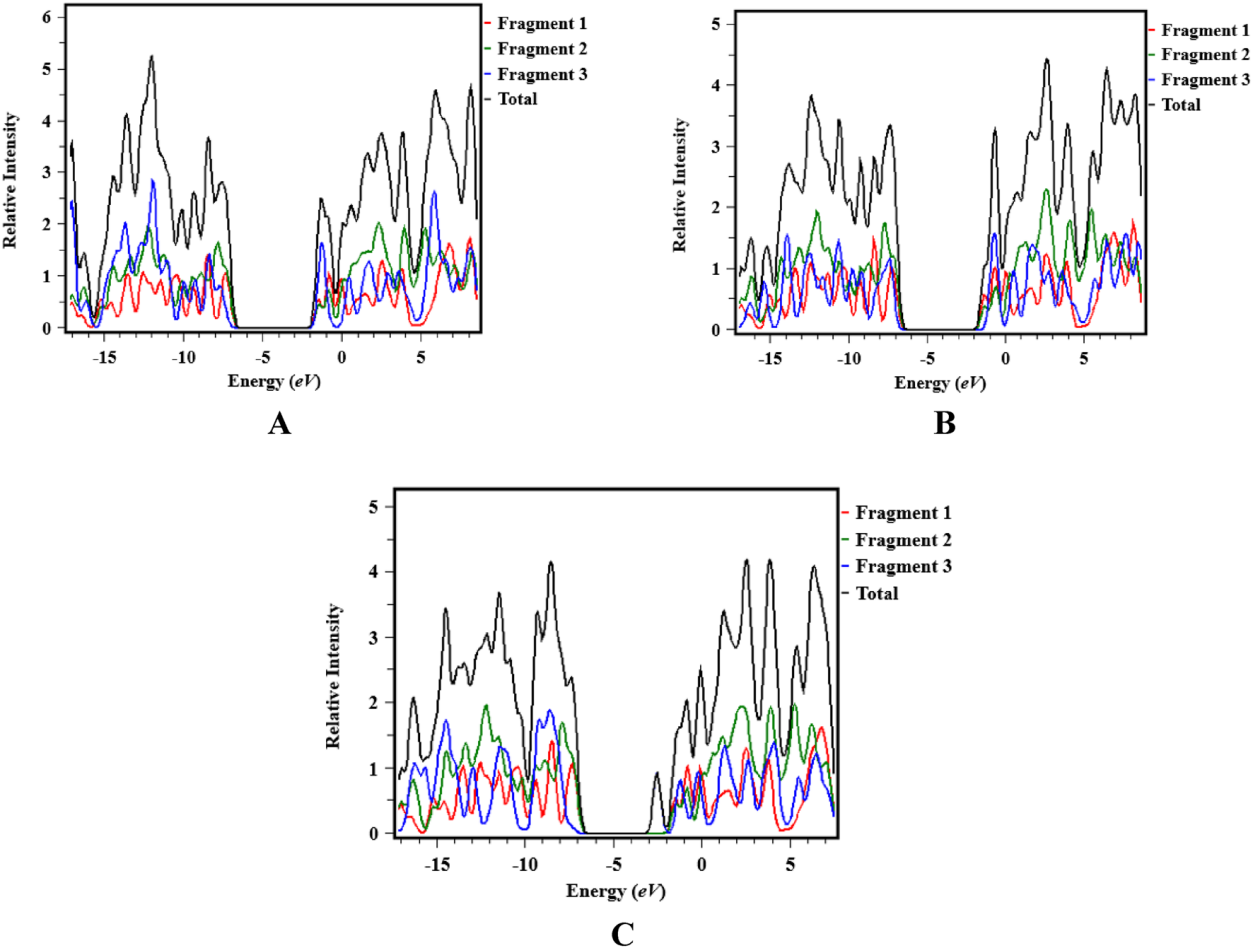
The DOS plots of the titled compounds (as segmented into three fragments: 1, 2, and 3 are shown with red, green, and blue lines, respectively).

The DOS further supports the findings of the FMO analysis by confirming electronic delocalization in the HOMO and LUMO orbitals^71^. Determining the distribution of electron charge on molecular orbitals by computing the DOS percentages for HOMOs and LUMOs offers additional support for the idea that the electron charge patterns are influenced by the differences in fragment 3. To comprehend the DOS, we segmented our chromophores into three components, namely, fragments 1, 2, and 3. The illustration in Fig. 4 depicts these fragments using red, green, and blue lines, correspondingly. Along the x-axis of DOS graphs, negative values denote the valence band (HOMO) whereas, positive values indicate the conduction band (LUMO). The energy gap is represented by the space between HOMO and LUMO^72^.

Figure 4 illustrates that the HOMO electron density of compound **A** is primarily located on fragment 3, while the LUMO charge density is predominantly distributed on fragment 3 and partially on fragment 2. In case of **B**, the HOMO charge density is mostly found on fragment 2, and fragment 3 having a small contribution, while the LUMOs are dispersed across fragment 2. For compound **C**, HOMOs are evenly distributed throughout fragments 2 and 3, but the LUMOs of the same compounds are located only on fragment 2. Table S9 demonstrates that for compounds **A**–**C**, fragment 1 contributes 46.8, 36.1, and 49.7% to HOMO and 53.5, 59.9, and 0.0% to LUMO, respectively, while fragment 2 contributes 51.5, 60.8, and 49.6% to HOMO and 40.8, 39.4, and 0.4% to LUMO, accordingly. Similarly, fragment 3 contributes 1.7, 3.1, and 0.7% to HOMO and 5.7, 0.7, and 99.6% to LUMO, respectively. The maximum HOMO charge density for compound **A** appears on fragment 2 at − 6.5 eV, while the highest LUMO charge density is found on fragment 3 at 1.5 eV, confirming an effective charge transfer from fragment 2 to fragment 3. In case of **B**, fragment 2 is found to have the greatest HOMO density at 7.2 eV whereas fragment 1 has the highest LUMO density. For **C,** fragment 1 depicts the highest maximum HOMO density of about 7.1 eV, whereas fragment 3 exhibit the highest maximum LUMO density of around 0.5 eV. This type of charge density favours the charge transfer from HOMO of fragment 1 to LUMO of fragment 3 via fragment 2. These findings demonstrate the importance of manipulating various components in fragment 3 to create diverse chromophores, influencing the transmission of electronic charges through distinct pathways. Overall, the results of DOS analysis significantly correlate with the outcomes of the FMO study.

### Transition density matrix (TDM) analysis

**Figure 5:**
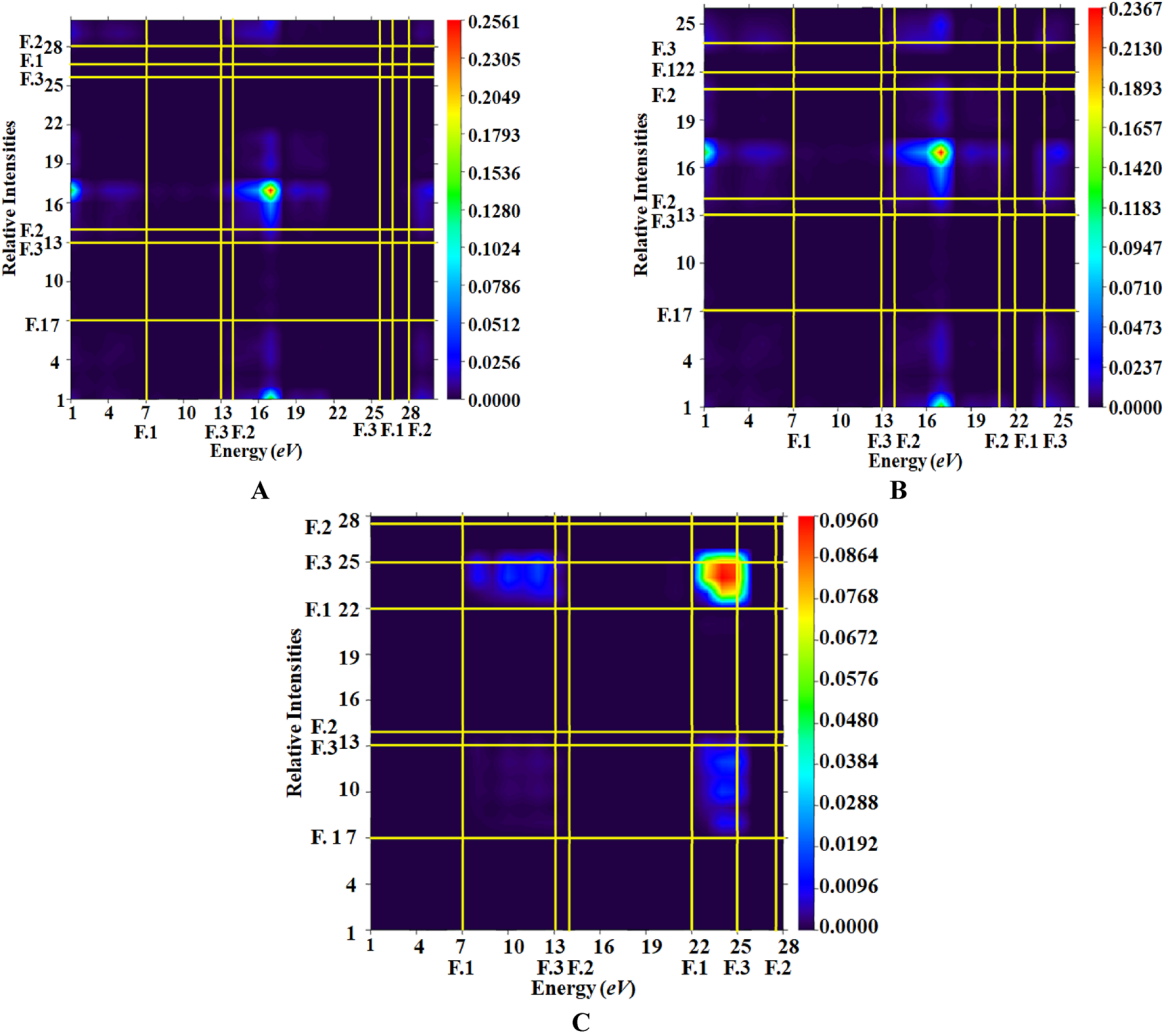
The TDM maps of compounds (**A**)–(**C**) (as segmented into three fragments: 1, 2, and 3 are shown diagonally charge transference between these segments).

The TDM is widely used to illustrate hole-electron pair dynamics and to explore nature of transitions in the examined compounds^73^. TDM analysis assists in the evaluation of electronic charge excitation processes, transitions from the ground state (S_0_) to excited state (S_1_), and electron–hole pair localization and delocalization. The TDM heat maps provide information about the nature of transition in these synthesized chromophores^47^. Figure 5 displays the transition density matrix (TDM) maps for all synthesized compounds, depicting the charge density transfer between the three distinct components (fragments 1, 2, and 3) of each chromophore. It is worth noting that the influence of hydrogen atoms on electronic transitions is not discussed in this study due to their minimal contribution.

Figure 5 depicts that in compounds **A** and **B**, the higher electronic cloud is clearly visible by green and red dots on fragment 3 and 2 of the heat maps. This electronic cloud exploited the successful transfer of electrons. However, compound **C** heat map shows that the electronic cloud is primarily on fragment 2, with a small amount of charge also visible on fragment 3. Such a pattern of electronic cloud facilitates the electron density to migrate towards fragment 3 without trapping. The presence of the very electron-deficient group (-NO_2_) on fragment 3 of compound **C** may be the reason for this peculiar behavior^74^. The TDM heat maps show efficient charge transfer coherence without any trapping in any of the investigated chromophores. These visual representations of TDM indicate a more straightforward and enhanced exciton dissociation in the excited state, facilitating the advancement of materials for NLO applications.

### Natural population analysis (NPA)

**Figure 6:**
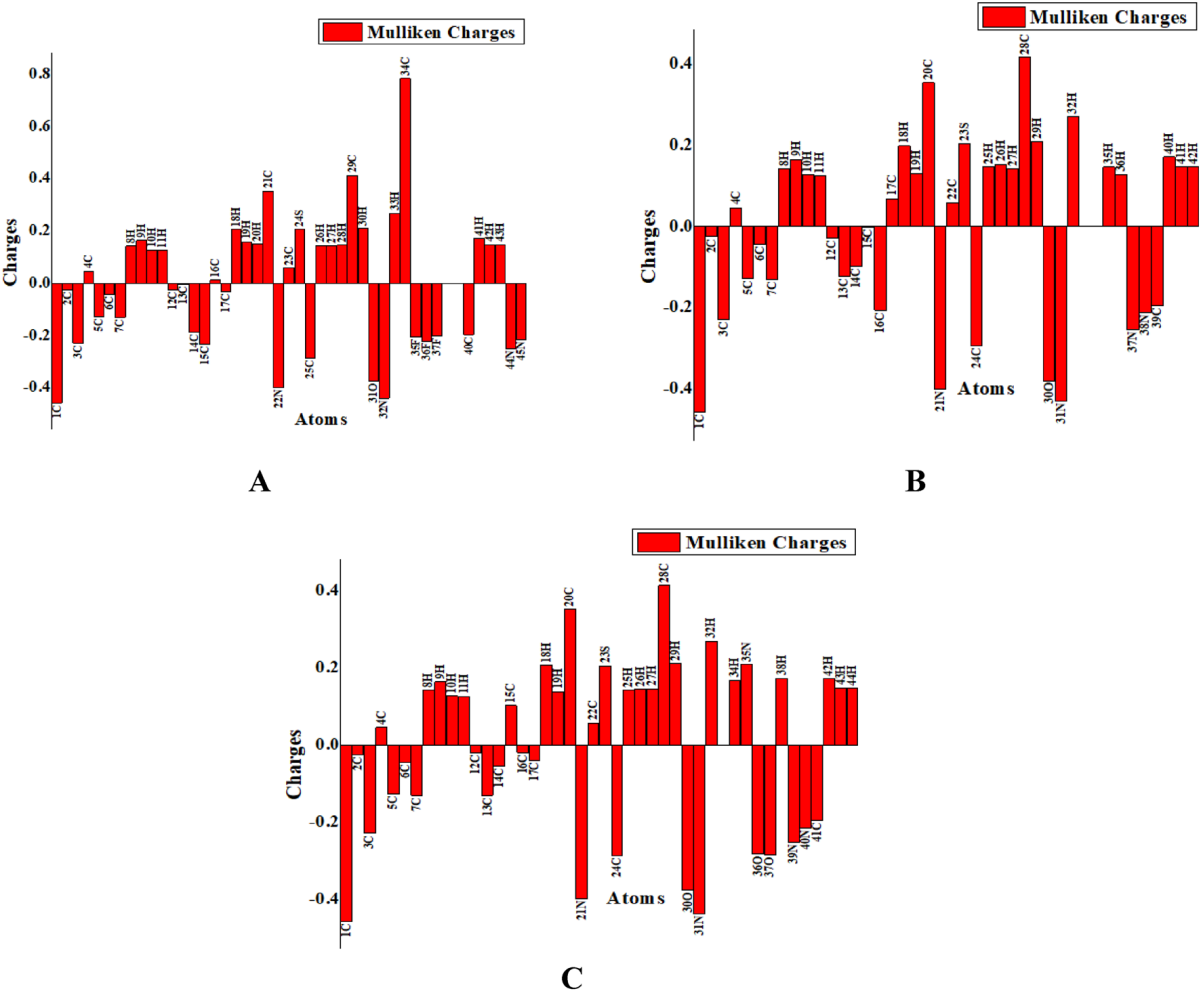
Natural population analysis graph of all the synthesized compounds illustrated the presence of electronegative elements.

The computation of effective atomic charges, which shows the distribution of positive and negative charges among atoms in the molecule, is essential for determining whether the length of the bonds between the atoms should increase or decrease. Atomic charges have an impact on the dipole moment, electrostatic potential surface, electronic structure, molecular polarizability, and numerous other properties of molecular systems^75,76^. Figure 6 demonstrates the results of an NPA analysis to determine the natural charges of **A**–**C** compounds. The process of charge transformation that results from reactions, as well as electrostatic potential on system surfaces are all commonly assessed using the natural charge examination^77^. The arrangement of molecules and their bonding capabilities are heavily impacted by the electrical charges carried by individual atoms. Natural charges of the molecules under investigation reveals that the presence of electronegative elements such as O, F, Cl, and Br are responsible for an uneven redistribution of the electron density across the benzene rings^78^. Additionally, Mulliken population analysis confirm that all of the hydrogen atoms have the same charge distribution, with the negative charges on carbon atoms leading to positive charges on hydrogen atoms.

From NPA, it can be observed that positive charges are present on all H, S, and Br-atoms. However, some C atoms have a positive charge while others have a negative charge. The presence of large negative charge on C atoms is due to their involvement in resonance with neighbouring N and O atoms. All N, Cl, and F-atoms were negatively charged except one nitrogen in compound **C**. Here it can be seen that C34 of compound **A**, C28 of compounds **B** and **C** showed a higher positive charge while N32 of compound **A**, N31 of compound **B** and **C** has a higher negative charge (see Fig. 6).

### Natural bond orbital (NBO) analysis

NBO analysis confirm the presence of hydrogen bonds resulting from hyper-conjugated interactions in the investigated chromophores^79^. Because all the orbitals are mathematically selected to include the highest possible percentage of the electron density, it provides the most precise representation of the natural Lewis structure. The NBO analysis effectively explains the interaction between the filled and virtual orbital space information^80^.

In Eq. (8), the stabilization energy E^(2)^ related to the delocalization *i → j* for each donor (*i*) and acceptor (*j*) is calculated by second-order perturbation theory^81^.

8 $${E}^{\left(2\right)}=\Delta {E}_{ij}={q}_{i}\frac{{\left({F}_{i,j}\right)}^{2}}{\left({E}_{j}-{E}_{i}\right)}$$

where *F*(*i. j*) is the off-diagonal NBO Fock matrix member, *i* and *j* are diagonal elements, and *q*_*i*_ is the donor orbital occupancy, while,* E*_i_ and *E*_j_ illustrate off-diagonal NBO Fock or Kohn–Sham medium elements ^82,83^. The stabilization energies of several orbitals have been examined and are presented in Tables S2–S4, while important transitions are depicted in Table 3.

**Table 3:** Chosen NBO analysis results for the examined chromophores (**A-C**).

Compounds	Donor (*i*)	Type	Acceptor (*j*)		Type	*E*(2) [*kcal/mol*]		*E*(*j*)-*E*(*i*) [*a.u.*]	F(*i,j*) [*a.u.*]	
**A**	C12-C17	*π*	C15-C16		π*	27.04		0.29	0.08	
	C5-C6	*π*	C21-N44		π*	15.42		0.29	0.059	
	N44-N45	*σ*	C23-S24		σ*	7.02		1.02	0.076	
	N22-C23	*σ*	C40-H43		σ*	0.51		1.2	0.022	
	N22	LP (1)	C21-N44		π*	48.4		0.31	0.111	
	F36	LP (1)	C15C34		σ*	0.5		1.47	0.025	
**B**	C13-C14	*π*	C12-C17		π*	24.87		0.3	0.077	
	C28-O30	*π*	C22-N38		π*	0.6		0.41	0.016	
	C2-C7	*σ*	C3-Br33		σ*	5.43		0.83	0.06	
	C17-N31	*σ*	N31-H32		σ*	0.54		1.2	0.023	
	N31	LP (1)	C28-O30		π*	55.33		0.33	0.121	
	S23	LP (2)	C39-H42		σ*	0.5		0.62	0.016	
**C**	C12-C13	*π*	C14-C15		π*	27.71		0.28	0.079	
	C28-O30	*π*	C22-N40		π*	0.63		0.41	0.016	
	N39-N40	*σ*	C22-S23		σ*	7.02		1.02	0.076	
	C13-H19	*σ*	C12-H18		σ*	0.57		0.96	0.021	
	O37	LP (3)	N35-O36		π*	179.33		0.16	0.154	
	S23	LP (2)	C12-C13		π*	0.54		0.28	0.012	

To enhance the interaction between the electron-donating and electron-accepting components, increased stabilization energies *E*^*(2)*^ are necessary. With increasing *E*^*(2)*^ value, the interaction between electron-deficient and electron-rich molecules becomes stronger. The studied compounds have six distinct transitions, namely π → π*, σ → σ*, π → σ*, σ → π*, LP → π*, and LP → σ*. However, the most prominent transitions are π → π*, σ → σ*, LP → π*, and LP → σ*. But the most dominant transitions are π → π*, followed by LP → π* and LP → σ*, while σ → σ* is the least dominant among all ^84^.

From Table 3, it is evident that in **A**, the greatest value of the most probable transition π(C12–C17) → π*(C15–C16) has been found to be 27.04 kcal/mol, the most dependable transition that results in the highest stabilization energy and a robust connection between donor (π) and acceptor (π*) units. π(C5–C6) to π*(C21–N44) transition, which had *E*^*(2)*^ value of 15.42 kcal/mol, was considered to be the least stable. Similarly, transitions like σ(N44–N45) → σ*(C23–S24) and σ(N22–C23) → σ*(C40–H43), have maximum and minimum *E*^*(2)*^ values of 7.02 and 0.51 kcal/mol, correspondingly. The outputs of these stabilization energies result from a weak interaction between π and π*. Electronic interactions such as LP1(N22) → π*(C21–N44) with the greatest stabilization energy value of 48.4 kcal/mol and LP1(F36) → σ*(C15–C34) with the lowest value of 0.5 kcal/mol were identified when the phenomena of resonance were taken into consideration.

The transition π(C13–C14) → π*(C12–C17) is the dominant π → π* transition depicted by **B** with *E*^*(2)*^ of 24.87 kcal/mol on the other hand, π(C28–O30) → π*(C22–N38) possess least *E*^*(2)*^ value at 0.6 kcal/mol. Due to enervated connections between the donor and acceptor, the transitions σ(C2–C7) → σ*(C3–Br33) and σ(C17–N31) → σ*(N31–H32) have the maximum and minimum energy values of 5.43 and 0.54 kcal/mol, correspondingly. In addition, resonance resulted in the stabilization energies of additional electronic transitions, such LP1(N31) → π*(C28–O30) and LP2(S23) → σ*(C39–H42) being 55.33 and 0.5 kcal/mol, respectively.

Data from Table 3 reveals that the compound C demonstrated that the transition π(C12–C13) → π*(C14–C15) had the least stabilization energy (0.63 kcal/mol) while the transition π(C28–O30) → π* (C22–N40) had the highest stabilization energy (27.71 kcal/mol). Weak interactions cause the transitions σ(N39–N40) → σ*(C22–S23) and σ(C13–H19) → σ*(C12–H18) to have senergies of 7.02 and 0.57 kcal/mol, correspondingly. The maximum energy discovered for LP → π* transition is 179.33 kcal/mol for LP3 (O37) → π*(N35–O36), while the lowest energy of LP → σ* is found to be 0.54 kcal/mol for LP2 (S23) → σ* (C12–C13).

### Nonlinear optical (NLO) properties

The development of materials with improved NLO behavior is required for the development of electro-optic modulation, frequency mixing, enhanced data rates, and harmonic generation related to the technology of potential communication^85,86^. Champagne and Bishop developed an intriguing topic by examining the effects of NLO on organic molecules^87^. Organic polymeric as well as heterocyclic compounds with significant hyper-polarizability amplitudes have attracted attention due to their potential use in NLO materials. Many research efforts develop effective methods for synthesizing organic chromophores in the NLO field because of their distinctive properties^88^. Compounds **A-C** have all been examined in the present study for possible NLO features by computational and experimental calculation. Based on the electronic characteristics, it is estimated that the intensity of the optical reactions would record a progressive linear as well as nonlinear responses^89^. The dipole moment (*µ*_*tot*_), polarizability < *α* > , first-order hyper-polarizability (*β*_tot_), and second-order hyper-polarizability (γ_*tot*_) values of the synthesized compounds computed by Eqs. (9)–(12) are shown in Table 4. Tables S5–S8 demonstrate the detailed outcomes for all tensors.

9 $$\mu = \, \left( {\mu_{x}^{2} + \mu_{y}^{2} + \mu_{z}^{2} } \right)^{{{1}/{2}}}$$

10 $$\left\langle \alpha \right\rangle = {1}/{3 }(\alpha_{xx} + \, \alpha_{yy} + \, \alpha_{zz} )$$

11 $$\beta_{{{\text{tot}}}} = (\beta_{(x)}^{2} + \beta_{y}^{2} + \beta_{z}^{2} )1/2$$

12 $$\gamma_{{{\text{tot}}}} = \sqrt {{\upgamma }_{x }^{2} + {\upgamma }_{y}^{2} + {\upgamma }_{z}^{2} }$$

**Table 4:** Computed dipole polarizability (***µ***_**tot**_) in Debye, average polarizability < ***α*** > , first hyperpolarizability (***β***_**tot**_) and second hyperpolarizability < γ > in esu of the **A-C** compounds.

Compounds	*µ*_tot_	< *α* > × 10^–23^	*β*_tot_ × 10^–30^	< γ > × 10^–35^
A	5.606	4.324	5.565	3.956
B	4.222	4.109	5.240	3.605
C	5.866	4.195	6.317	4.314

$${\text{Where\,}} {\gamma }_{i}= \frac{1}{15 }\sum_{j}({\gamma }_{ijji}+{\gamma }_{ijij}+{\gamma }_{iijj})\, i,j =$$

An essential consideration for determining the polarizability of organic chromophores is the dipole moment (*µ*_*tot*_)^90^ which is the measure of the separation of charges. The *µ*_*tot*_ of chromophores **A**–**C** is found to be 5.606, 4.222, and 5.866 *D*, respectively. Compound **C** has the highest total value of all the compounds (5.866 *D*), which may be due to the presence of a potent electron-withdrawing group NO_2_. On the other side, **B** which has a weak electron withdrawing chloro group in fragment 3, has the lowest value of dipole moment at 4.222 *D*. The overall increasing order of dipole moment is **B < A < C**. The prominent values of *µ*_*y*_ (4.697, 3.718, and 5.066 *D*, respectively), shown in Table S5, suggest that the greater polarity for compounds **A**–**C** resides along the y-axis (ordinate). Furthermore, on comparing the outcomes with standard para-nitroaniline chromophore (*µ*_para-nitroaniline_ = 6.3 *D*), it is observed that the newly synthesized compounds exhibit better polarity than standard p-NA.

The average polarizability was observed to be the highest in compound **A** (4.324 × 10^–23^ esu). The lowest value of < ***α*** > , on the other hand, was found in **B** (4.109 × 10^–23^ esu). This was because of the presence of the CF_3_ group along with the chloro group that enriched the electron density and improved the electron-withdrawing capacity. The < ***α*** > values of **A**–**C** are 4.324 × 10^–23^, 4.109 × 10^–23^, and 4.195 × 10^–23^ esu, accordingly and tensors of < ***α*** > are duslpayed in Table S6. According to the literature, molecule polarizability is influenced by the HOMO–LUMO energy gap^91^. The HOMO–LUMO energy gap is inversely related to both linear and nonlinear polarizabilities. Small HOMO–LUMO energy gap compounds enable high nonlinear and linear polarizabilities. In contrast to the energy gap values for **A**, **B**, and **C**, a small energy gap for **C** is seen in our analysis. As a result, its linear and nonlinear polarizability values are higher.

Among all of the developed compounds, the highest value of *β*_tot_ was observed in **C** at 6.317 × 10^–30^ esu as it has a powerful electron-withdrawing unit − NO_2_. A slight decrease in *β*_tot_ value was found in **A** i.e., 5.565 × 10^–30^ esu*.* However, the lowest value of *β*_*tot*_ was observed in **B** (5.240 × 10^–30^ esu). First-order hyper-polarizability responses of **A**–**C** were 401.5616, 1605.112, and 1298.838 times greater than that of the *p-NA* (3.610 × 10^–34^), respectively.

For each of the investigated chromophores, a notable secondary hyperpolarizability was observed in the y-axis direction, as illustrated in Table S7, with **C** having a greater value of γ_*tot*_ among the derivatives. The decreasing order of second-order hype-polarizability (γ_*tot*_) values is shown as: **C > A > B**. In a comparative investigation, we also studied the non-linear optical characteristics of *p-NA* (3.812 × 10^–36^) used as the standard chromophore. This comparison with *p-NA* demonstrates that compounds **A**–**C**, with compound** C** being the most effective among them, are efficient candidates for NLO materials. The reasoning above also leads to the conclusion that introducing different groups in fragment 3 results in yielding significant NLO amplitude.

## Conclusion

In the current research, synthesis of three novel N-substituted 2-((5-(3-bromophenyl)-4-methyl-4H-1,2,4-triazol-3-yl)thio)-N-phenylpropanamide derivatives have been reported in multiple step approach. The structures of these compounds were analyzed by spectroscopic analysis. These spectral analyses provided crucial evidence supporting the structural integrity of the synthesized compound. All synthesized hybrids **(7a–7c)** exhibited UV–Vis absorption spectrum within the range of 298 nm, 297 nm, and 295 nm. IR spectra confirmed the presence of specific functional groups peaks associated with NH groups 3272–3158 cm^−1^, carbonyl groups (C=O) 1733–1677 cm^−1^, N=C 1694–1641 cm^−1^, and N=N 1596–1526 cm^−1^, and aromatic C=C stretching 1480–1440 cm^−1^ while NMR spectra detected quartets, singlets, and doublets, at 4.58–4.82, 3.55–3.65, and 1.71–1.75 ppm, and H-4′ and H-5′ contained triplets at 7.39–7.67 ppm in all compounds’ spectra **(7a**–**7c)**. According to the FMO data, compound **C** had the lowest band gap of 4.618 eV. Additionally, global reactivity parameters were also correlated to the band gap, compound **C** exhibits the lowest global hardness of 2.309 eV and the highest softness value of 0.216 eV. Furthermore, higher values of NBO-based hyper conjugative interactions were noted, endorsing the highest stability of the molecules under investigation. The charge distribution and electrophilic and nucleophilic areas of the examined chromophores were explained by natural population analysis According to TDM heat maps, the reduced band gap will help the effective transmission of charges from fragment 2 to fragment 3. DOS pictographs provide more support to this efficient transfer of electronic cloud. Moreover, the synthesised compound **C** exhibits a notable large NLO response with values of 5.866 *D*, 4.195, 6.317, and 4.314 esu for *µ*_tot_, < *α* > , (*β*_tot_) and < γ > , respectively. Based on the FMO data, compound C exhibits the lowest band gap, highest stability, lowest global hardness, and highest softness values. Furthermore, its reduced band gap and notable large NLO response suggest its suitability for applications that require efficient charge transmission and strong NLO properties. These findings contribute to the understanding of the structure–property relationships and pave the way for further exploration and utilization of these compounds in optoelectronic and related applications.

### Computational procedure

At first the structure of yielded compounds were optimized at M06/6-311G(d,p) functional with the aid og Gussian 09 software. After getting the true minima geometries of **A**–**C** chromophores, different kind of analyse like FMOs, NPA, NLO, DOS and TDM were accomplished at DFT/TDDFT approached by utilizing above-mentional level. Different software like PyMOlyze 2.0 program Origin^92^ and Gauss Sum^93^ were utilized to interprete the data from jobs.

### Supplementary Information

Supplementary Information.

## Data Availability

All data generated or analyzed during this study are included in this published article and its supplementary information files.
