# Supplementary material for: Exploration of nonlinear optical properties of 4-methyl-4H-1,2,4-triazol-3-yl)thio)-N-phenylpropanamide based derivatives: experimental and DFT approach
Source: Sci Rep. 2024 Feb 1;14:2732. doi: 10.1038/s41598-024-51788-z (PMC10834427; doi:10.1038/s41598-024-51788-z)
Supplement: Supplementary file 1 — Supplementary Information. [file 41598_2024_51788_MOESM1_ESM.docx]

**Supplementary Data**

**Exploration of Nonlinear Optical Properties of 4-methyl-4H-1,2,4-triazol-3-yl)thio)-*N*-phenylpropanamide Based Derivatives: Experimental and DFT Approach**

Muhammad Irfan,^1^ Hammad Ali Khan,^1^ Shamsa Bibi,^2^ Gang Wu,^3^ Akbar Ali,^1^ Samreen Gul Khan,^*1^ Norah Alhokbany,^4^ Faiz Rasool*^5^, Ke Chen,*^3^

^1^Department of Chemistry, Faculty of Physical Science, Government College University, Faisalabad-38000, Pakistan.

^2^Department of Chemistry, University of Agriculture, Faisalabad, Pakistan.

^3^Department of Infectious Diseases, The Affiliated Hospital of Southwest Medical University, Luzhou 646000, China

^4^Department of Chemistry, College of Science, King Saud University, Riyadh, 11451, Saudi Arabia.

^5^Institute of Chemical Sciences, Bahauddin Zakariya University, Multan, 60800, Pakistan

*Corresponding author's E-mail addresses:

Dr. Samreen Gul Khan: [samreengul@gcuf.edu.pk](mailto:samreengul@gcuf.edu.pk)

Dr. Faiz Rasool (faizrasoolbzu@gmail.com)

Dr. Ke Chen: chitty8705@sina.com


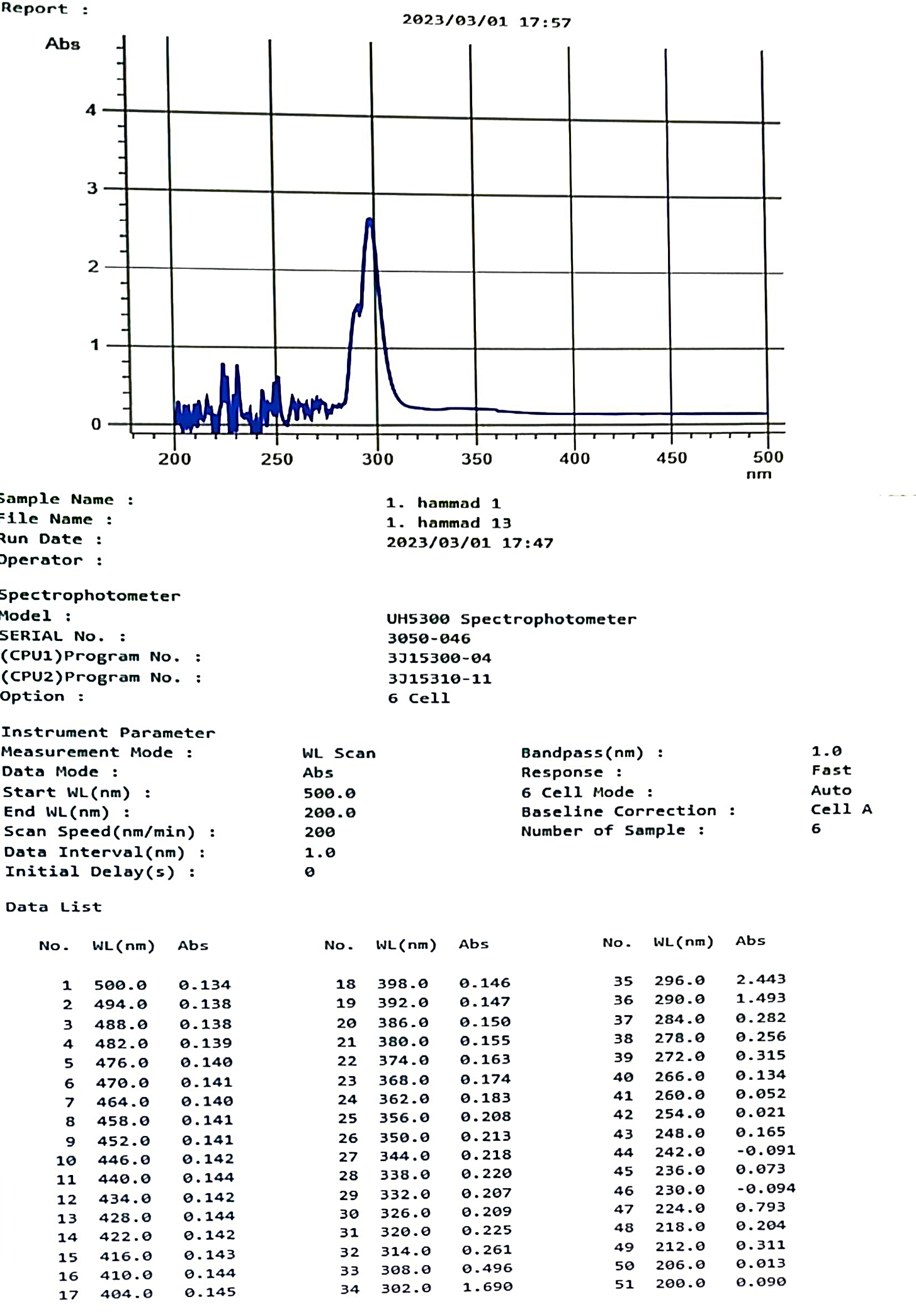


**Figure S1:** UV-Visible spectrum of compound **7a**


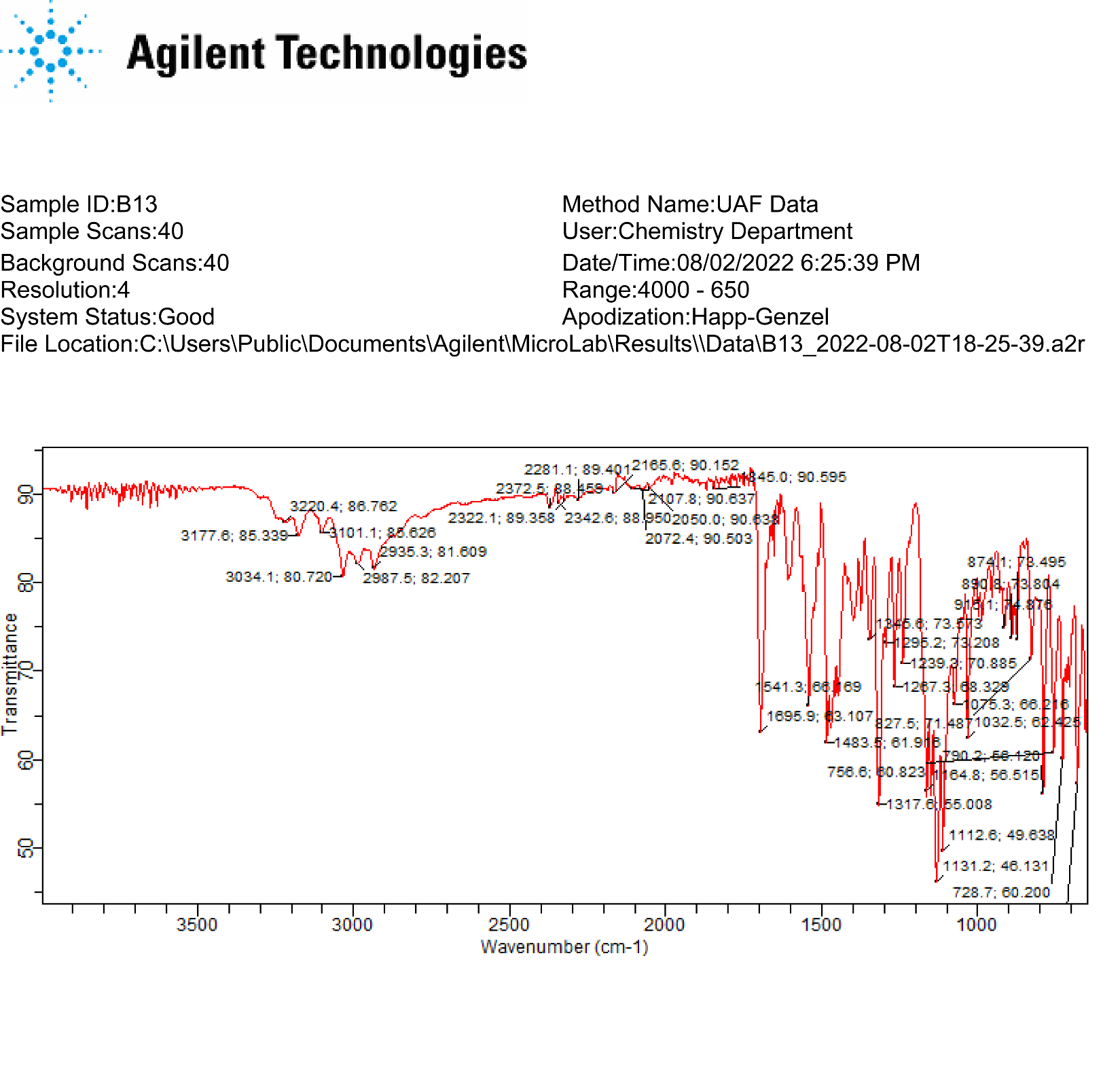


**Figure S 2:** IR spectrum of compound **7a**

**Figure S 3:** 1H NMR spectrum of compound **7a (Full spectrum)**


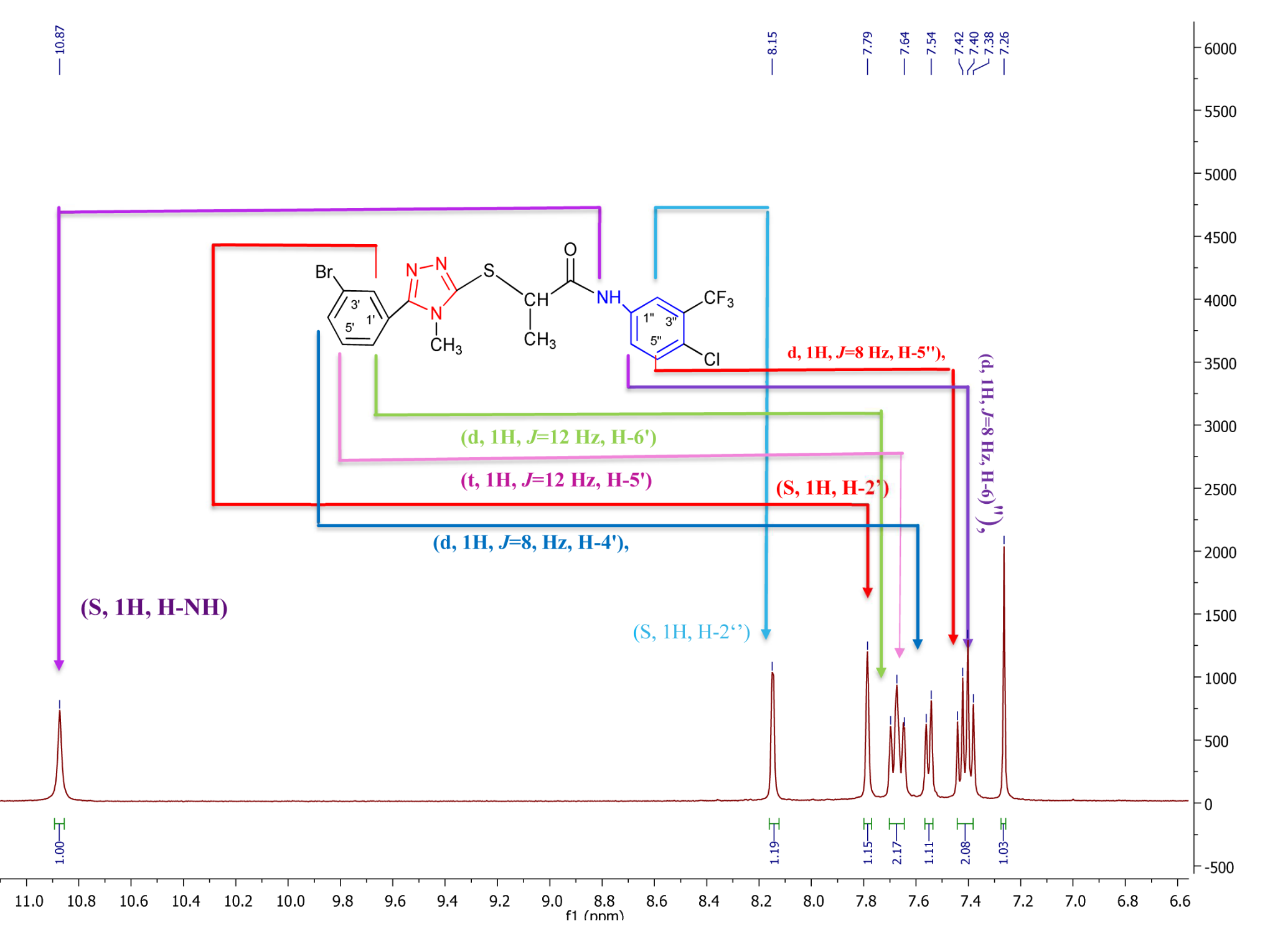


**Figure S 4:** ^1^H NMR spectrum of compound **7a (Aromatic region)**


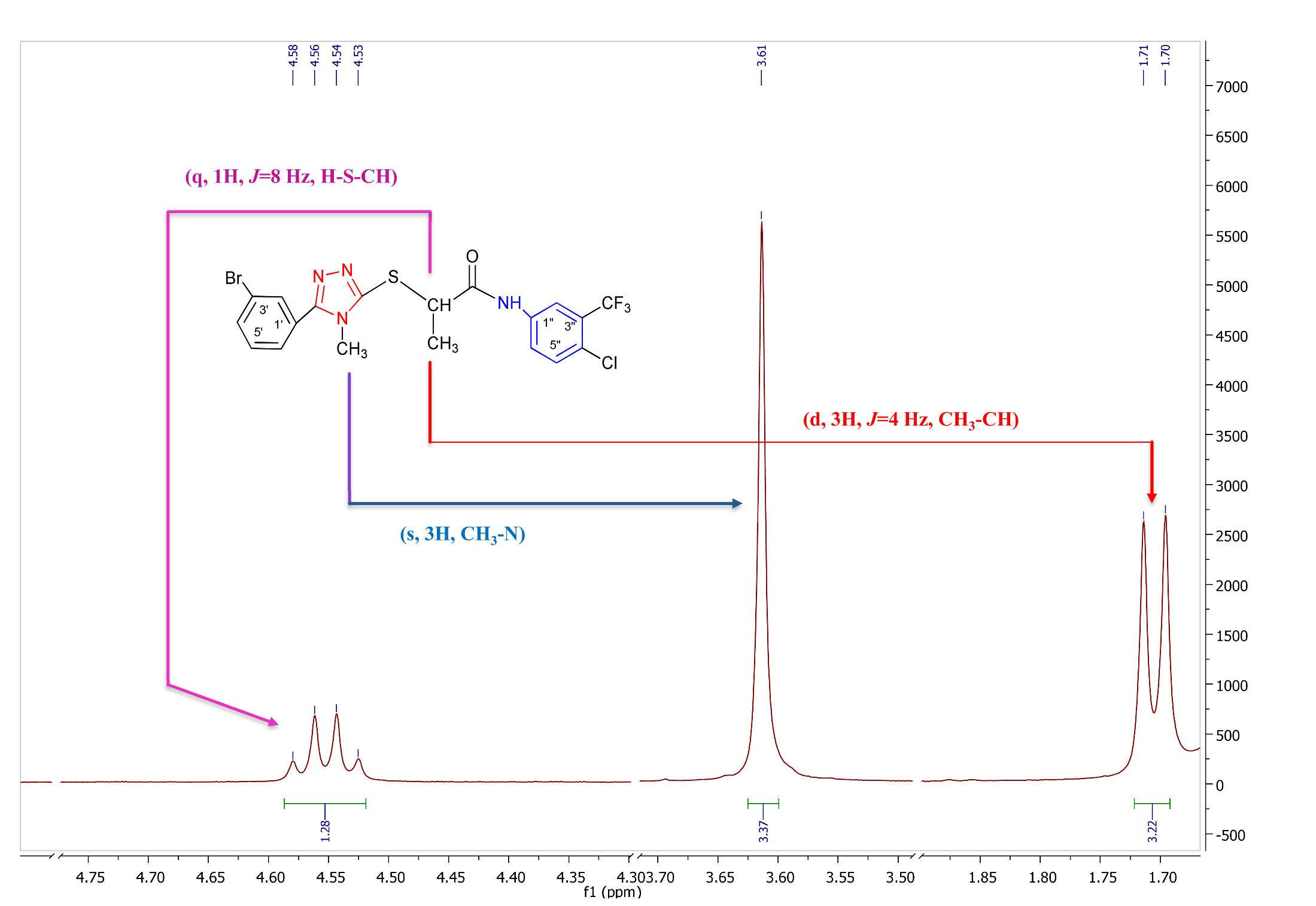


**Figure S5**: ^1^H NMR spectrum of compound **7a (Aliphatic region)**


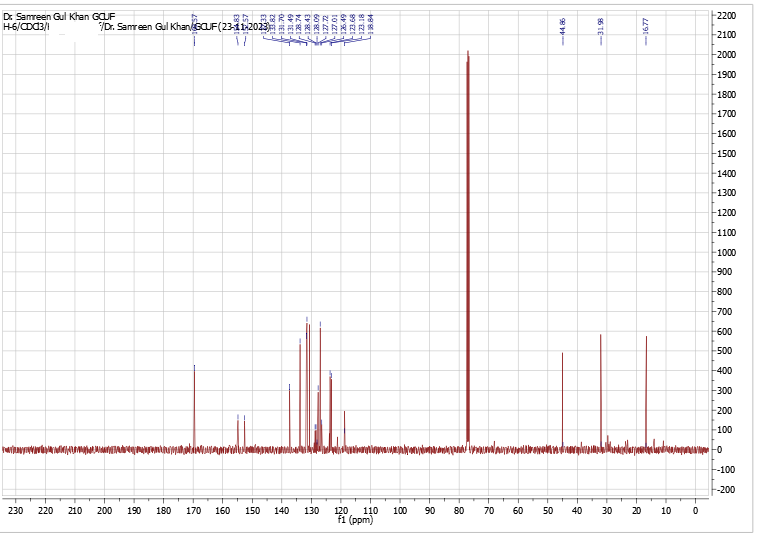


***Figure S6****:* ***^13^C NMR*** *spectrum of compound* ***of 7a***


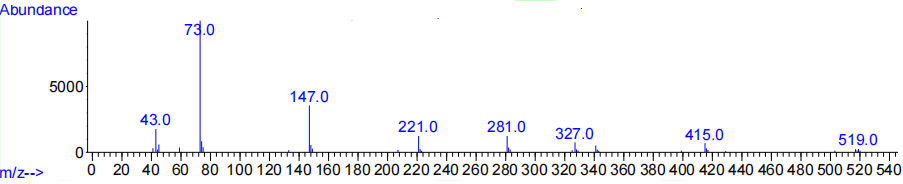


***Figure S7****:* ***GCMS Analysis*** *of compound* ***of 7a***


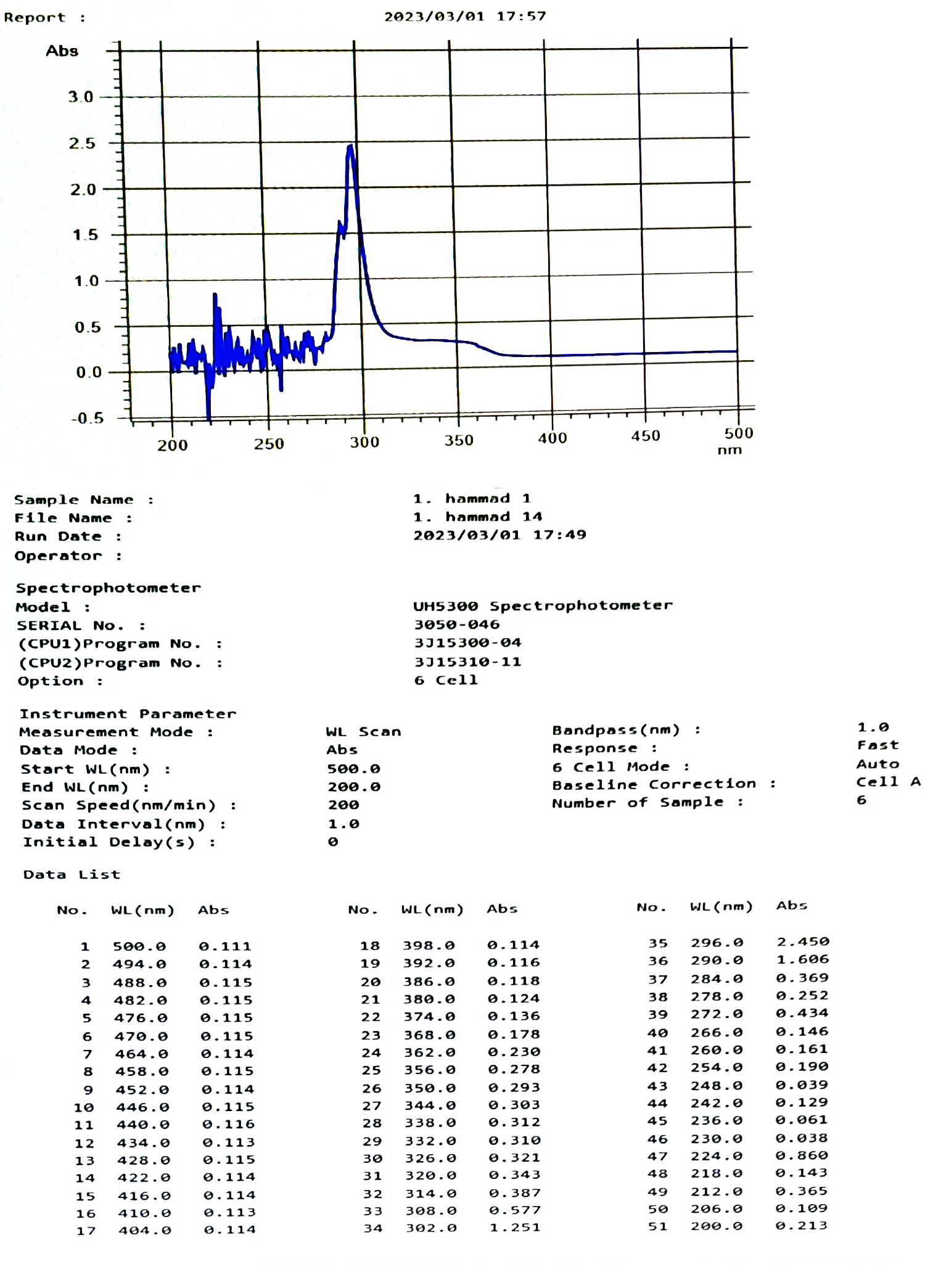


**Figure S 8;** UV-Visible spectrum of compound **7b**


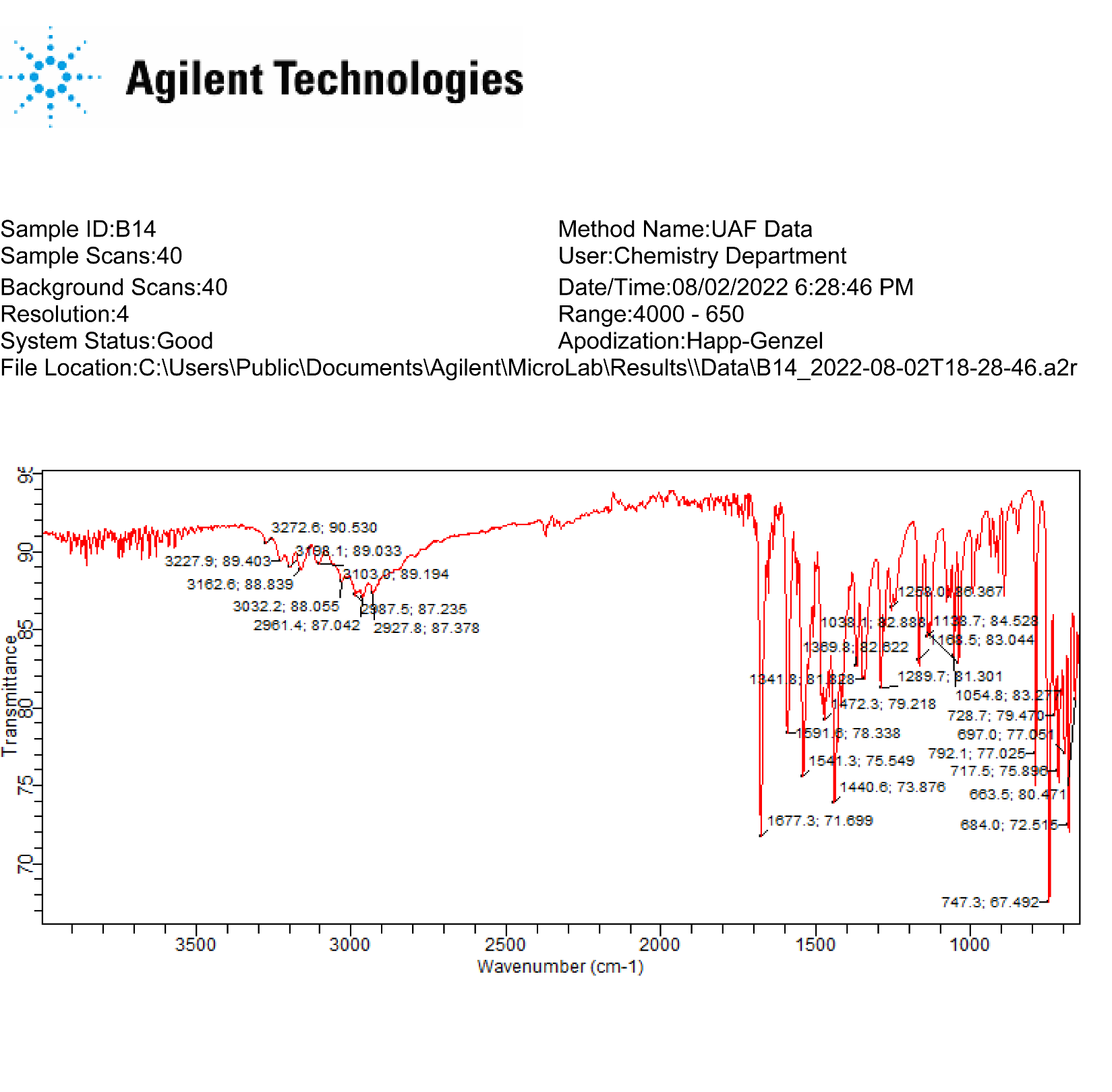


**Figure S9:** IR spectrum of compound **7b**

**Figure S 10:** ^1^H NMR spectrum of compound **7b (Full spectrum)**


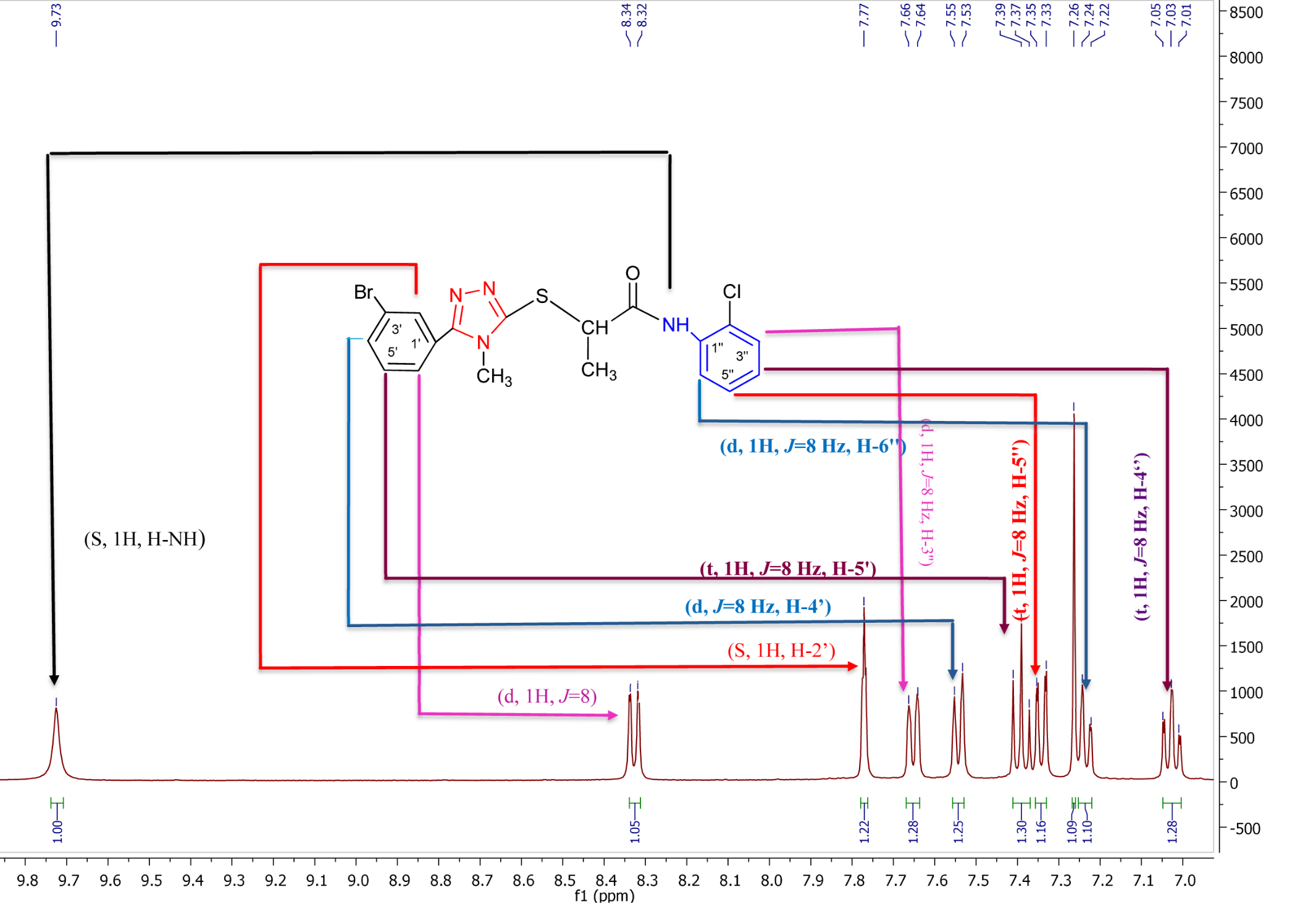


**Figure S 11**: ^1^H NMR spectrum of compound **7b (Aromatic region)**


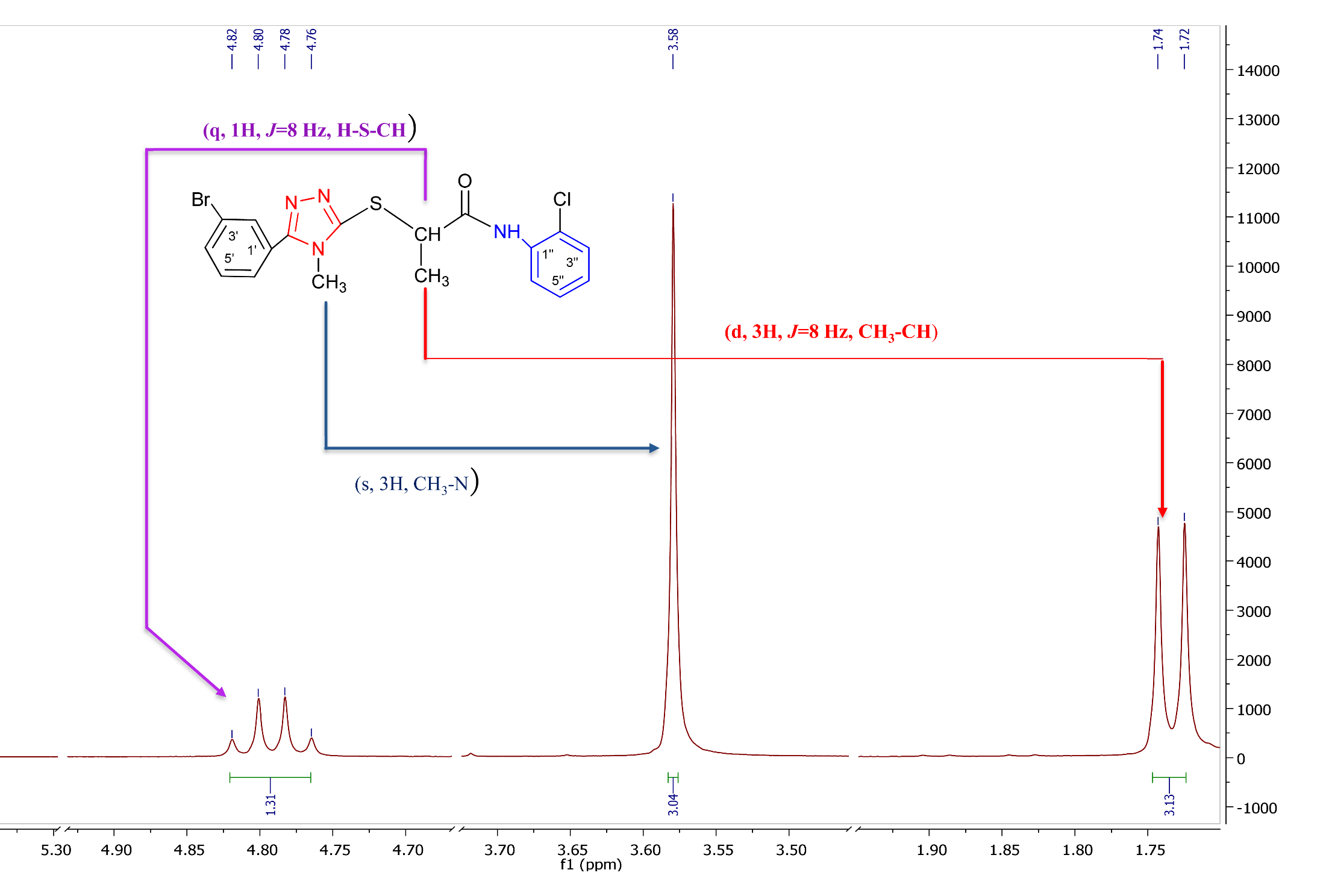


**Figure S12:** ^1^H NMR spectrum of compound **7b (Aliphatic region)**


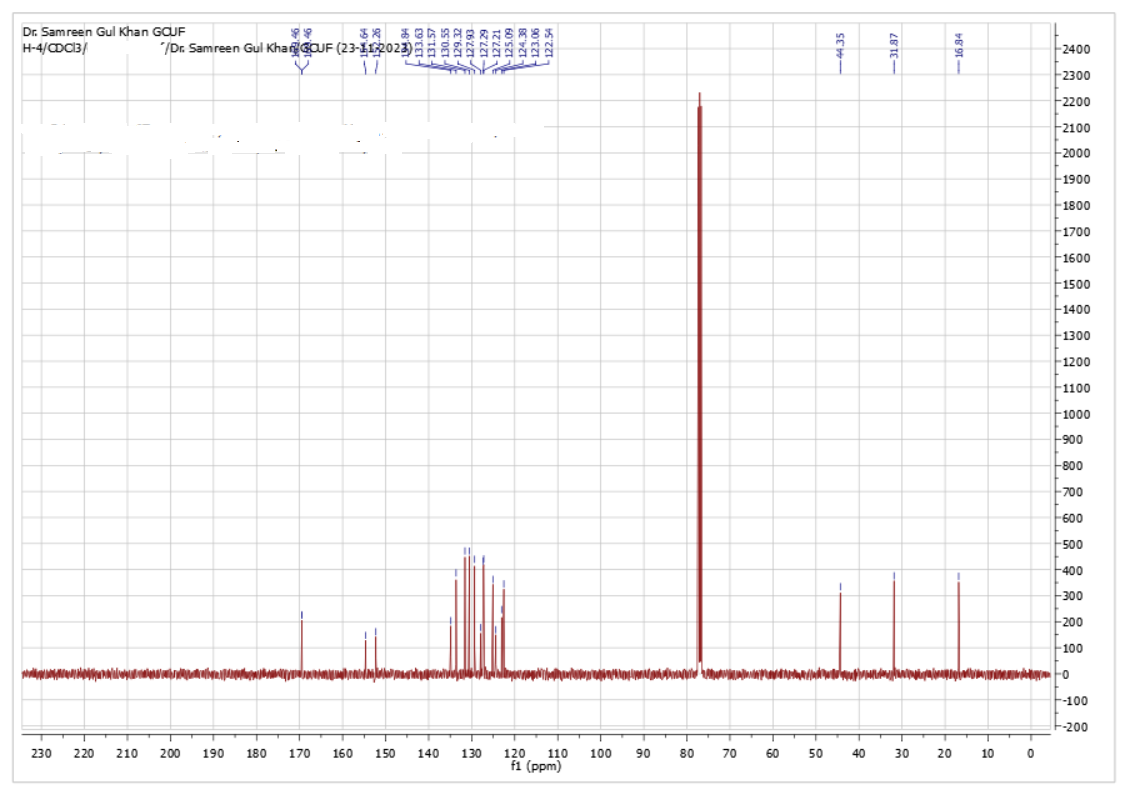


***Figure S13****:* ***^13^C NMR*** *spectrum of compound* ***of 7b***


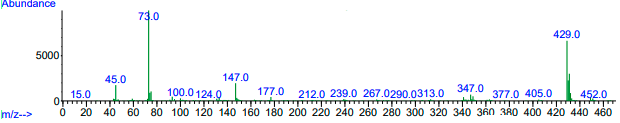


***Figure S14****:* ***GCMS Analysis*** *of compound* ***of 7b***


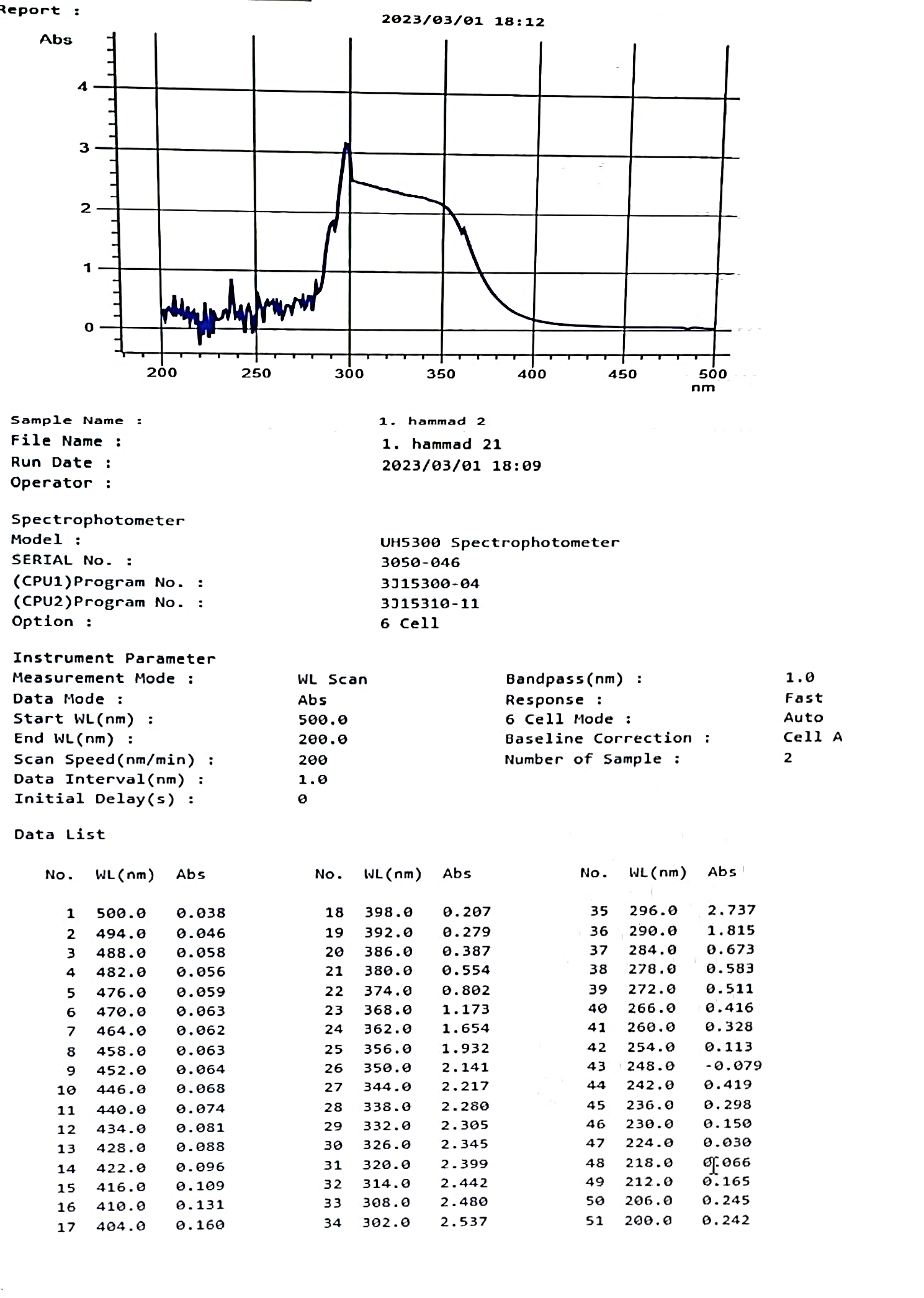


**Figure S 15** : UV-Visible spectrum of compound **7c**


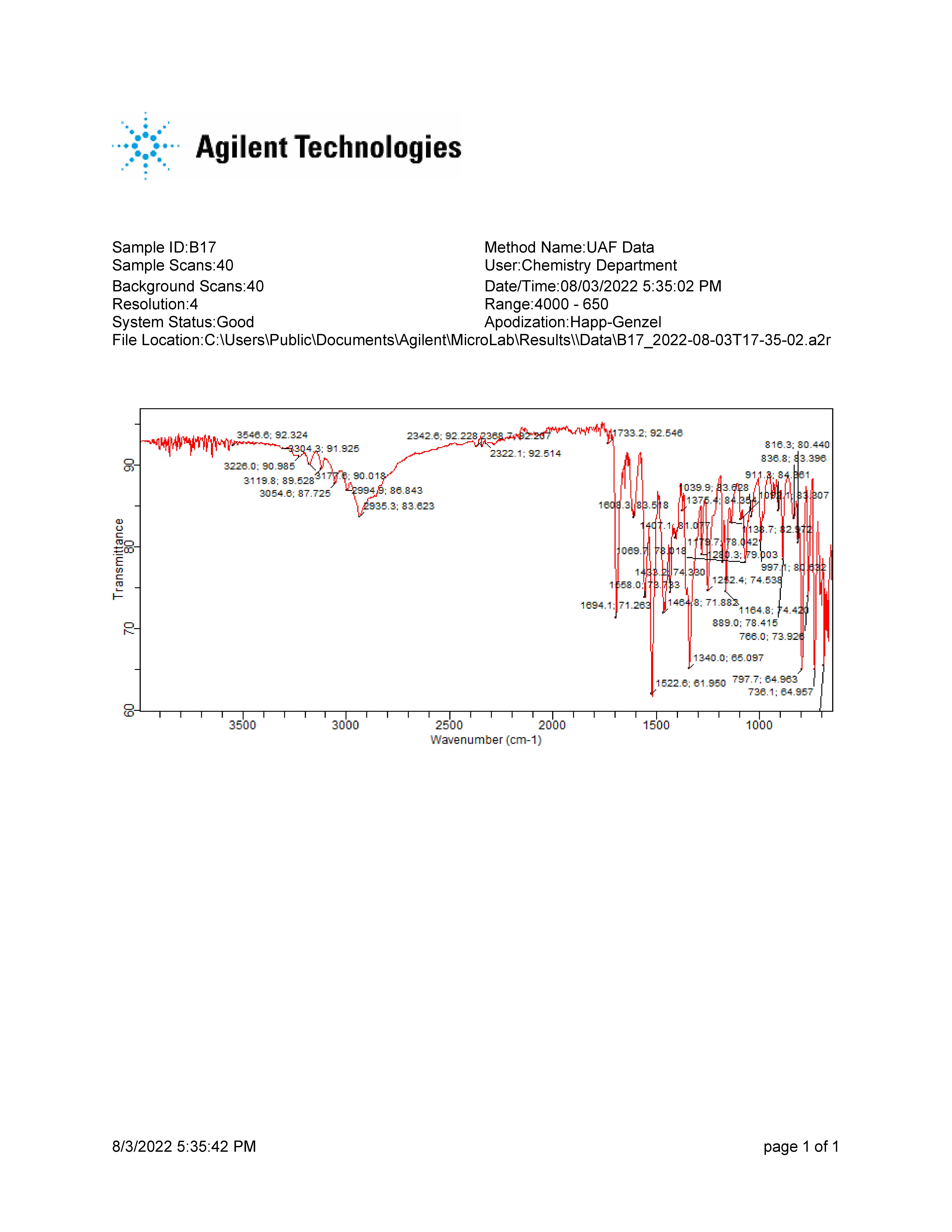


**Figure S16**: IR spectrum of compound **7c**

**Figure S17:** ^1^H NMR spectrum of compound **7c (Full spectrum**)


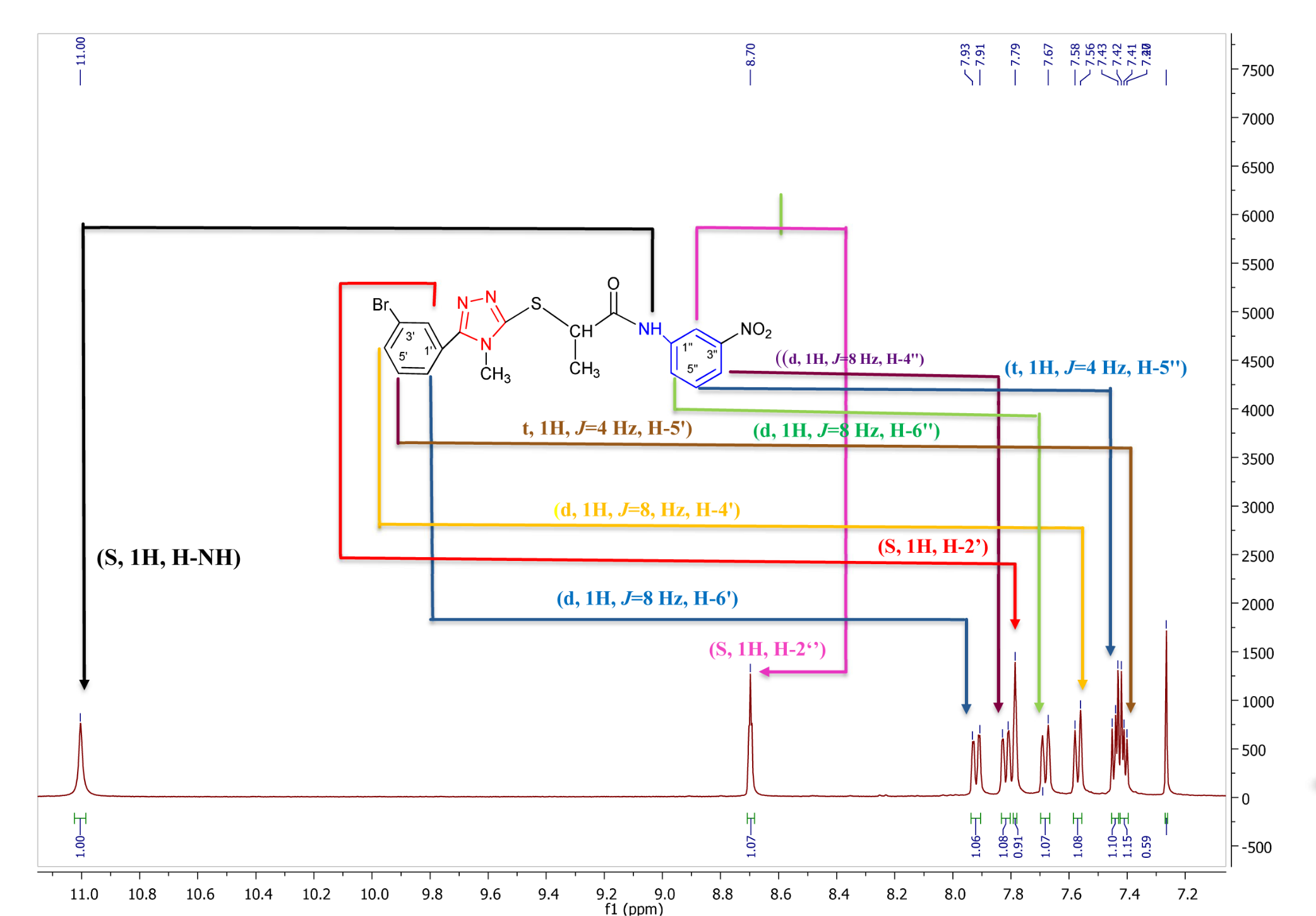


**Figure S18**: ^1^H NMR spectrum of compound **7c (Aromatic region)**


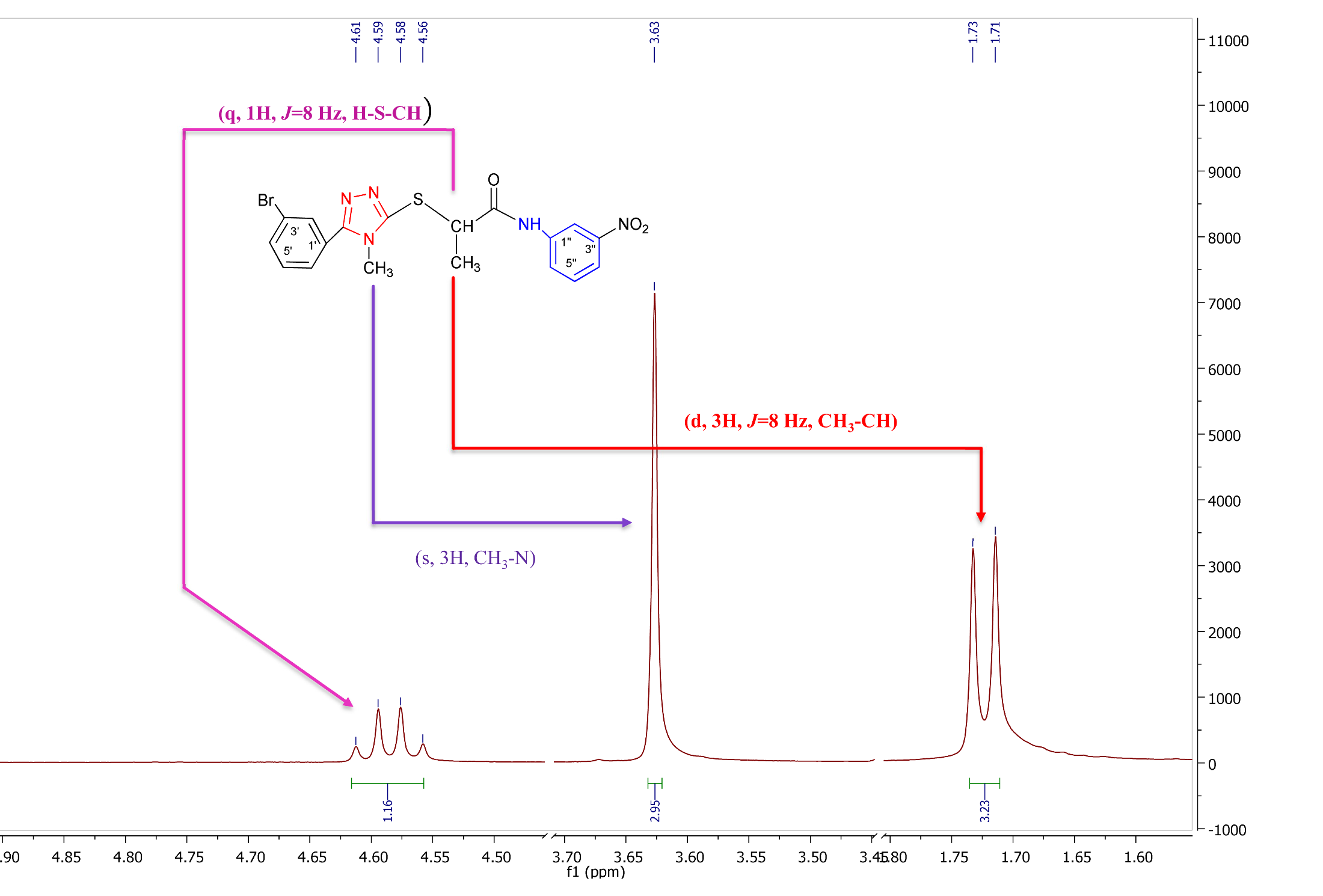


**Figure S19:** ^1^H NMR spectrum of compound **7c (Aliphatic region)**


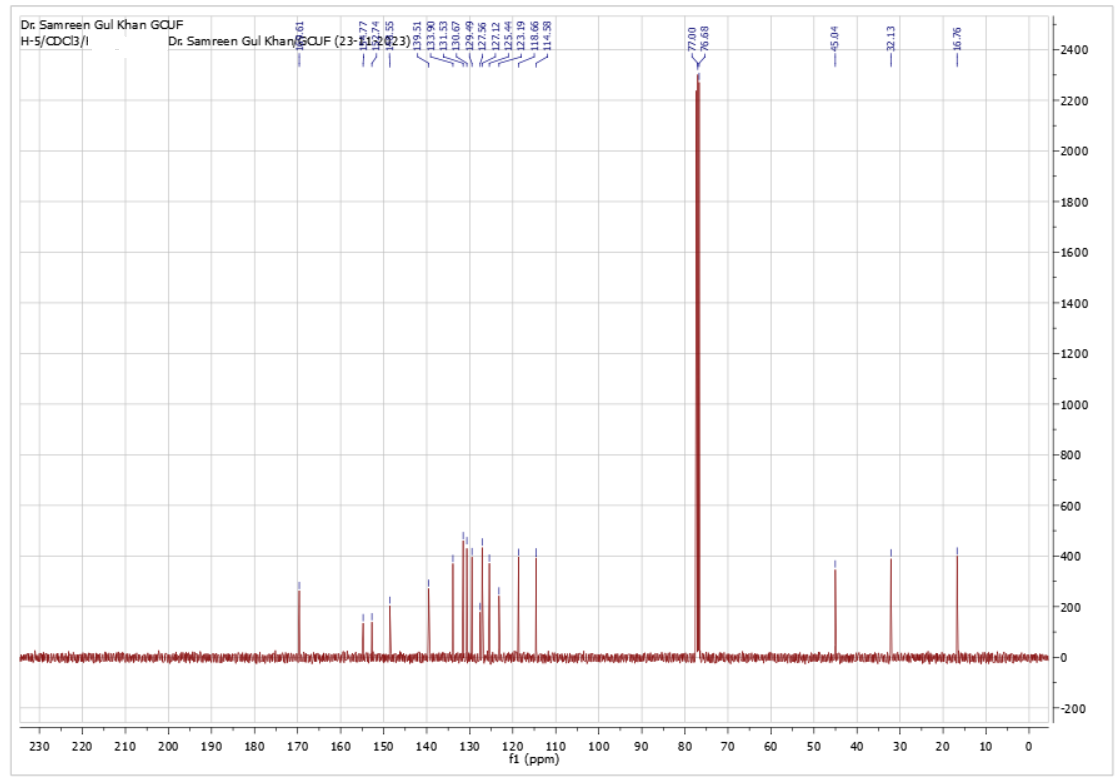


***Figure S20****:* ***^13^C NMR*** *spectrum of compound* ***of 7c***


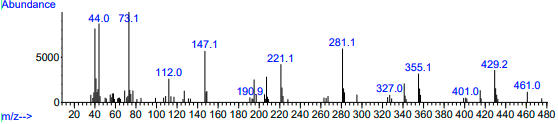


***Figure S21****:* ***GCMS Analysis*** *of compound* ***of 7c***

| **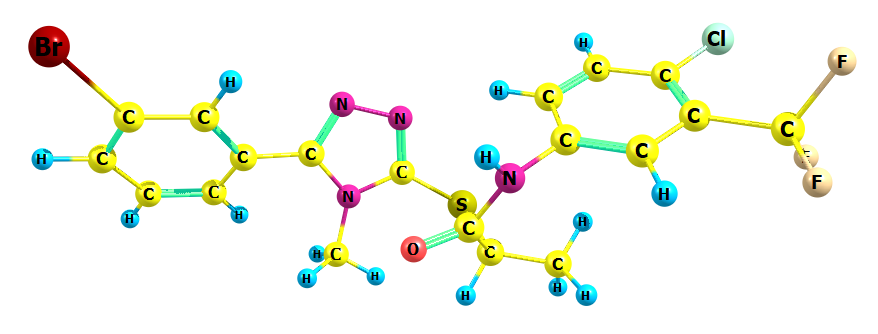** | **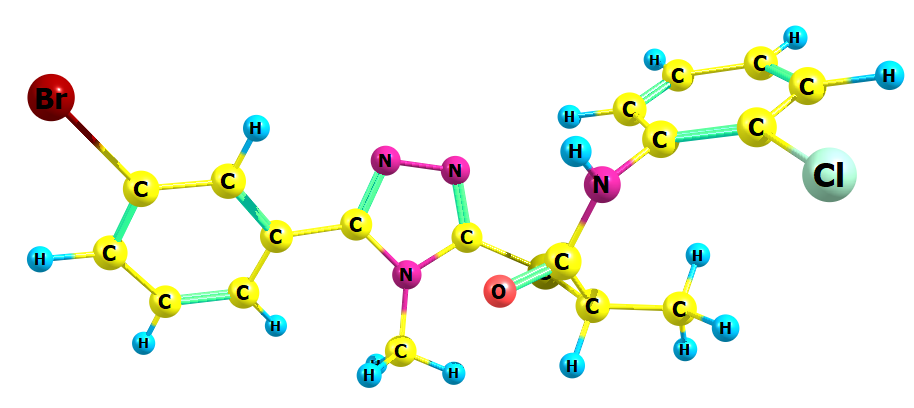** |
| --- | --- |
| **A** | **B** |
| **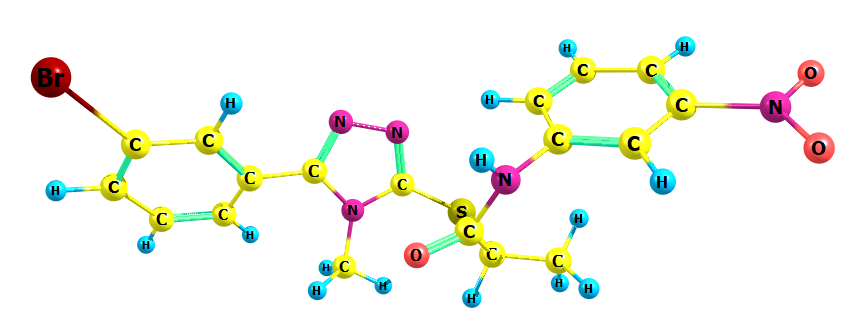** | |
| **C** | |

**Figure S22: Optimized structures of A-C.**

**Table S1**: Calculated energies (*E*) and energy gap (∆*E*) of HOMO-1, LUMO+1, HOMO-2 and LUMO+2 for **A-C.**

| **Compounds** | **HOMO-1** | **LUMO+1** | **∆*E*** | **HOMO-2** | **LUMO+2** | **∆*E*** |
| --- | --- | --- | --- | --- | --- | --- |
| **A** | -7.304 | -1.336 | 5.968 | -7.472 | -1.192 | 6.28 |
| **B** | -7.140 | -0.880 | 6.26 | -7.348 | -0.686 | 6.662 |
| **C** | -7.337 | -1.497 | 5.84 | -7.498 | -1.218 | 6.28 |

*E*=energy, ∆*E*(e*V*)=*E*_LUMO_-*E*_HOMO_; HOMO=highest occupied molecular orbital; LUMO=lowest unoccupied molecular orbital, MO=molecular orbital, Units in *eV*

**Table S2:** Natural bond orbitals analysis for **A** with its representative values.

| **Donor(*i*)** | **Type** | **Acceptor(*j*)** | **Type** | ***E^(2^)***  **[*kcal/mol*]** | ***E(j)-E(i)***  **[*a.u*]** | ***F(i,j)***  **[*a.u*]** |
| --- | --- | --- | --- | --- | --- | --- |
| C12-C17 | π | C15-C16 | π* | 27.04 | 0.29 | 0.08 |
| C12-C17 | π | C13-C14 | π* | 21.94 | 0.29 | 0.071 |
| C5-C6 | π | C3-C4 | π* | 22.43 | 0.3 | 0.073 |
| C2-C7 | π | C3-C4 | π* | 21.96 | 0.29 | 0.072 |
| C13-C14 | π | C15-C16 | π* | 20.72 | 0.31 | 0.072 |
| C3-C4 | π | C2-C7 | π* | 22.05 | 0.31 | 0.074 |
| C15-C16 | π | C12-C17 | π* | 18.27 | 0.31 | 0.068 |
| C3-C4 | π | C5-C6 | π* | 19.86 | 0.31 | 0.071 |
| C5-C6 | π | C21-N44 | π* | 15.42 | 0.29 | 0.059 |
| C21-N44 | π | C5-C6 | π* | 7.58 | 0.36 | 0.049 |
| N44-N45 | σ | C23-S24 | σ* | 7.02 | 1.02 | 0.076 |
| C2-C3 | σ | C3-C4 | σ* | 4.26 | 1.33 | 0.067 |
| C15-C16 | σ | C14-C15 | σ* | 6.7 | 1.28 | 0.083 |
| C7-H11 | σ | C5-C6 | σ* | 4.29 | 1.1 | 0.061 |
| C25-H27 | σ | C1-S24 | σ* | 5.22 | 0.64 | 0.052 |
| C12-C13 | σ | C14-Cl38 | σ* | 4.96 | 0.87 | 0.059 |
| C12-H18 | σ | C13-C14 | σ* | 4.12 | 1.09 | 0.06 |
| C7-H11 | σ | C2-C7 | σ* | 0.72 | 1.11 | 0.025 |
| N32-H33 | σ | C12-C17 | σ* | 0.61 | 1.23 | 0.024 |
| C13-C14 | σ | C12-H18 | σ* | 2.17 | 1.18 | 0.045 |
| C16-H20 | σ | C14-C15 | σ* | 4.81 | 1.08 | 0.065 |
| C12-H18 | σ | C16-C17 | σ* | 4.88 | 1.1 | 0.065 |
| C12-C17 | σ | C12-C13 | σ* | 3.42 | 1.31 | 0.06 |
| C5-C6 | σ | C4-C5 | σ* | 5.12 | 1.29 | 0.072 |
| C1-C29 | σ | C29-N32 | σ* | 0.92 | 1.14 | 0.029 |
| C13-C14 | σ | C12-C13 | σ* | 3.68 | 1.33 | 0.063 |
| C14-C15 | σ | C13-H19 | σ* | 2.03 | 1.15 | 0.043 |
| C29-N32 | σ | C29-O31 | σ* | 1.15 | 1.43 | 0.036 |
| C34-F37 | σ | C34-F35 | σ* | 0.57 | 1.34 | 0.025 |
| N22-C23 | σ | C40-H43 | σ* | 0.51 | 1.2 | 0.022 |
| N22 | LP (1) | C21-N44 | π* | 48.4 | 0.31 | 0.111 |
| N32 | LP (1) | C12-C17 | π* | 0.89 | 0.31 | 0.015 |
| Cl38 | LP (3) | C13-C14 | π* | 14.76 | 0.34 | 0.068 |
| Br39 | LP (3) | C3-C4 | π* | 10.55 | 0.32 | 0.056 |
| S24 | LP (1) | C23-N45 | π* | 1.25 | 0.65 | 0.028 |
| F35 | LP (2) | C15-C16 | π* | 0.77 | 0.47 | 0.018 |
| O31 | LP (2) | C29-N32 | σ* | 25.97 | 0.73 | 0.125 |
| F36 | LP (3) | C34-F35 | σ* | 10.85 | 0.72 | 0.08 |
| N45 | LP (1) | N22-C23 | σ* | 8.32 | 0.83 | 0.074 |
| N44 | LP (1) | C23-N45 | σ* | 7.28 | 0.94 | 0.074 |
| Cl38 | LP (2) | C13-C14 | σ* | 4.32 | 0.9 | 0.056 |
| S24 | LP (2) | C1-C29 | σ* | 1.45 | 0.64 | 0.027 |
| S24 | LP (2) | N22-C23 | σ* | 8.94 | 0.68 | 0.07 |
| F36 | LP (1) | C15C34 | σ* | 0.5 | 1.47 | 0.025 |

**Table S3:** Natural bond orbitals analysis for **B** with its representative values.

| **Donor(*i*)** | **Type** | **Acceptor(*j*)** | **Type** | ***E^(2^)***  **[*kcal/mol*]** | ***E(j)-E(i)***  **[*a.u*]** | ***F(i,j)***  **[*a.u*]** |
| --- | --- | --- | --- | --- | --- | --- |
| C13-C14 | π | C12-C17 | π* | 24.87 | 0.3 | 0.077 |
| C5-C6 | π | C3-C4 | π* | 22.42 | 0.29 | 0.073 |
| C2-C7 | π | C3-C4 | π* | 21.92 | 0.29 | 0.072 |
| C3-C4 | π | C5-C6 | π* | 19.81 | 0.31 | 0.071 |
| C20-N37 | π | C22-N38 | π* | 18.8 | 0.32 | 0.074 |
| C5-C6 | π | C20-N37 | π* | 15.47 | 0.29 | 0.06 |
| C28-O30 | π | C28-O30 | π* | 0.88 | 0.47 | 0.019 |
| C12-C17 | π | C15-C16 | π* | 24.06 | 0.29 | 0.075 |
| C22-N38 | π | C20-N37 | π* | 14.23 | 0.36 | 0.068 |
| C20-N37 | π | C5-C6 | π* | 7.75 | 0.35 | 0.05 |
| C28-O30 | π | C22-N38 | π* | 0.6 | 0.41 | 0.016 |
| C15-C16 | π | C13-C14 | π* | 20.14 | 0.32 | 0.072 |
| C2-C7 | σ | C3-Br33 | σ* | 5.43 | 0.83 | 0.06 |
| C5-C6 | σ | C4-C5 | σ* | 5.09 | 1.29 | 0.072 |
| C4-H9 | σ | C2-C3 | σ* | 4.76 | 1.09 | 0.064 |
| C2-C3 | σ | C3-C4 | σ* | 4.26 | 1.33 | 0.067 |
| C5-C20 | σ | C20-N21 | σ* | 2.19 | 1.13 | 0.045 |
| C2-H8 | σ | C6-C7 | σ* | 3.78 | 1.11 | 0.058 |
| C4-C5 | σ | C5-C20 | σ* | 3.31 | 1.19 | 0.056 |
| C5-C20 | σ | C4-C5 | σ* | 3.1 | 1.26 | 0.056 |
| C12-C13 | σ | C14-H36 | σ* | 2.53 | 1.13 | 0.048 |
| C14-C15 | σ | C13-H19 | σ* | 2.37 | 1.14 | 0.046 |
| C15-C16 | σ | C15-H35 | σ* | 1.62 | 1.15 | 0.039 |
| C12-C17 | σ | C12-H18 | σ* | 1.4 | 1.15 | 0.036 |
| C14-C15 | σ | C15-H35 | σ* | 1.39 | 1.12 | 0.035 |
| C24-H27 | σ | C1-C28 | σ* | 4.62 | 0.9 | 0.058 |
| C15-H35 | σ | C15-C16 | σ* | 1.1 | 1.1 | 0.031 |
| C13-H19 | σ | C12-C13 | σ* | 0.84 | 1.11 | 0.027 |
| C14-H36 | σ | C13-H19 | σ* | 0.78 | 0.95 | 0.024 |
| C17-N31 | σ | N31-H32 | σ* | 0.54 | 1.2 | 0.023 |
| N31 | LP (1) | C28-O30 | π* | 55.33 | 0.33 | 0.121 |
| N21 | LP (1) | C20-N37 | π* | 48.46 | 0.31 | 0.111 |
| Cl34 | LP (3) | C15-C16 | π* | 13.71 | 0.35 | 0.067 |
| S23 | LP (1) | C22-N38 | π* | 1.29 | 0.65 | 0.028 |
| N31 | LP (1) | C12-C17 | π* | 1.69 | 0.31 | 0.021 |
| Br33 | LP (3) | C3-C4 | π* | 10.49 | 0.32 | 0.056 |
| S23 | LP (2) | C22-N38 | π* | 0.74 | 0.25 | 0.013 |
| O30 | LP (2) | C28-N31 | σ* | 25.87 | 0.73 | 0.125 |
| N38 | LP (1) | N21-C22 | σ* | 8.35 | 0.82 | 0.074 |
| N37 | LP (1) | C20-N21 | σ* | 8.23 | 0.84 | 0.074 |
| Br33 | LP (2) | C2-C3 | σ* | 3.53 | 0.88 | 0.05 |
| N37 | LP (1) | C22-N38 | σ* | 7.25 | 0.94 | 0.074 |
| Cl34 | LP (2) | C15-C16 | σ* | 4.16 | 0.9 | 0.055 |
| N38 | LP (1) | C12-H18 | σ* | 2.4 | 0.84 | 0.04 |
| N31 | LP (1) | C28-O30 | σ* | 1.51 | 0.86 | 0.034 |
| S23 | LP (2) | C39-H42 | σ* | 0.5 | 0.62 | 0.016 |

**Table S4:** Natural bond orbitals analysis for **C** with its representative values.

| **Donor(*i*)** | **Type** | **Acceptor(*j*)** | **Type** | ***E^(2^)***  **[*kcal/mol*]** | ***E(j)-E(i)***  **[*a.u*]** | ***F(i,j)***  **[*a.u*]** |
| --- | --- | --- | --- | --- | --- | --- |
| C12-C13 | π | C14-C15 | π* | 27.71 | 0.28 | 0.079 |
| C14-C15 | π | C16-C17 | π* | 24.83 | 0.31 | 0.078 |
| C16-C17 | π | C12-C13 | π* | 22.83 | 0.31 | 0.076 |
| C2-C7 | π | C3-C4 | π* | 21.96 | 0.29 | 0.072 |
| C16-C17 | π | C14-C15 | π* | 20.65 | 0.3 | 0.071 |
| C3-C4 | π | C5-C6 | π* | 19.86 | 0.31 | 0.071 |
| C5-C6 | π | C20-N39 | π* | 15.42 | 0.29 | 0.059 |
| N35-O36 | π | C14-C15 | π* | 2.98 | 0.49 | 0.038 |
| C3-C4 | π | C2-C7 | π* | 22.04 | 0.31 | 0.074 |
| C20-N39 | π | C5-C6 | π* | 7.56 | 0.36 | 0.049 |
| C28-O30 | π | C22-N40 | π* | 0.63 | 0.41 | 0.016 |
| N39-N40 | σ | C22-S23 | σ* | 7.02 | 1.02 | 0.076 |
| C20-N21 | σ | C22-S23 | σ* | 6.26 | 1.03 | 0.072 |
| C24-H26 | σ | C1-S23 | σ* | 5.25 | 0.64 | 0.052 |
| C41-H44 | σ | C20-N21 | σ* | 5.29 | 0.99 | 0.065 |
| C24-H27 | σ | C1-C28 | σ* | 4.67 | 0.9 | 0.059 |
| C20-N39 | σ | N21-C41 | σ* | 4.26 | 1.17 | 0.063 |
| C4-H9 | σ | C2-C3 | σ* | 4.75 | 1.09 | 0.064 |
| C22-N40 | σ | N21-C41 | σ* | 4.15 | 1.2 | 0.063 |
| C14-H34 | σ | C12-C13 | σ* | 3.86 | 1.12 | 0.059 |
| C13-C14 | σ | C12-C13 | σ* | 3.16 | 1.31 | 0.058 |
| C5-C6 | σ | C6-C7 | σ* | 3.7 | 1.3 | 0.062 |
| C3-Br33 | σ | C2-C7 | σ* | 3.13 | 1.25 | 0.056 |
| C3-C4 | σ | C5-C20 | σ* | 3.01 | 1.23 | 0.054 |
| C1-S23 | σ | C24-H26 | σ* | 2.72 | 0.96 | 0.046 |
| C1-C28 | σ | N31-H32 | σ* | 2.27 | 1.07 | 0.044 |
| C2-C3 | σ | C7-H11 | σ* | 2.2 | 1.15 | 0.045 |
| C4-H9 | σ | C3-C4 | σ* | 1.51 | 1.11 | 0.036 |
| C2-C7 | σ | C7-H11 | σ* | 1.19 | 1.13 | 0.033 |
| C2-H8 | σ | C2-C3 | σ* | 1.15 | 1.1 | 0.032 |
| C6-H10 | σ | C6-C7 | σ* | 0.95 | 1.12 | 0.029 |
| C7-H11 | σ | C2-H8 | σ* | 0.76 | 0.93 | 0.024 |
| C13-H19 | σ | C12-H18 | σ* | 0.57 | 0.96 | 0.021 |
| O37 | LP (3) | N35-O36 | π* | 179.33 | 0.16 | 0.154 |
| N21 | LP (1) | C20-N39 | π* | 48.42 | 0.31 | 0.111 |
| S23 | LP (1) | C22-N40 | π* | 1.25 | 0.65 | 0.028 |
| S23 | LP (2) | C12-C13 | π* | 0.54 | 0.28 | 0.012 |
| O30 | LP (2) | C28-N31 | σ* | 25.98 | 0.73 | 0.125 |
| O37 | LP (2) | N35-O36 | σ* | 20.59 | 0.76 | 0.113 |
| O36 | LP (2) | C15-N35 | σ* | 15.6 | 0.58 | 0.085 |
| N31 | LP (1) | C12-C17 | σ* | 7.23 | 0.85 | 0.075 |
| N40 | LP (1) | N21-C41 | σ* | 0.65 | 0.72 | 0.02 |
| N31 | LP (1) | C16-C17 | σ* | 5.66 | 0.85 | 0.066 |
| S23 | LP (1) | N21-C22 | σ* | 2.38 | 1.08 | 0.046 |
| O30 | LP (1) | C28-N31 | σ* | 1.44 | 1.16 | 0.037 |
| S23 | LP (1) | C22-N40 | σ* | 0.81 | 1.2 | 0.028 |
| N39 | LP (1) | C5-C20 | σ* | 0.59 | 0.88 | 0.02 |

**Table S5**: Dipole moment and major contributing tensors (*a.u*) of the studied compounds (**A-C**).

| **Dipole moment** | **A** | **B** | **C** |
| --- | --- | --- | --- |
| *µ_x_* | -2.581 | 1.643 | -2.883 |
| *µ_y_* | 4.697 | 3.718 | 5.066 |
| *µ_z_* | -1.642 | -1.141 | -0.666 |
| *µ_total_* | 5.606 | 4.222 | 5.866 |

**Table S6**: Polarizability and major contributing tensor (*esu*) of the studied compounds (**A-C**).

| **Polarizability** | **A** | **B** | **C** |
| --- | --- | --- | --- |
| *α_xx_* | 6.162×10^-23^ | 5.589×10^-23^ | 5.957×10^-23^ |
| *α_yy_* | 3.756×10^-23^ | 3.600×10^-23^ | 3.660×10^-23^ |
| *α_zz_* | 3.053×10^-23^ | 3.140×10^-23^ | 2.967×10^-23^ |
| *α_total_* | 4.324×10^-23^ | 4.109×10^-23^ | 4.195×10^-23^ |

**Table S7**: Second hyperpolarizability and major contributing tensor (*esu*) of the studied compounds (**A-C**).

| **2^nd^ Hyper pol.** | **A** | **B** | **C** |
| --- | --- | --- | --- |
| *γ_X_* | 2.992×10^-35^ | 2.738×10^-35^ | 3.300×10^-35^ |
| *γ_Y_* | 6.793×10^-36^ | 5.766×10^-36^ | 7.639×10^-36^ |
| *γ_Z_* | 2.852×10^-36^ | 2.904×10^-36^ | 2.499×10^-36^ |
| *Average* <*γ*> | 3.956×10^-35^ | 3.605×10^-35^ | 4.314×10^-35^ |
| *Magnitude of γ* | 3.081×10^-35^ | 2.813×10^-35^ | 3.397×10^-35^ |

**Table S8**: Frequency-dependent first hyperpolarizability (*esu*) of studied compounds (**A-C**).

| **Polarizability** | **A2** | **B2** | **C2** |
| --- | --- | --- | --- |
| ***β_xxx_*** | 2.590×10^-30^ | 1.048×10^-30^ | -2.671×10^-30^ |
| ***β_xxy_*** | -1.106×10^-30^ | -1.188×10^-30^ | 4.865×10^-31^ |
| ***β_xyy_*** | -9.742×10^-31^ | -1.126×10^-30^ | -1.836×10^-30^ |
| ***β_yyy_*** | -3.487×10^-30^ | -3.346×10^-30^ | -3.008×10^-30^ |
| ***β_xxz_*** | 1.166×10^-30^ | 9.256×10^-31^ | 2.038×10^-30^ |
| ***β_yyz_*** | 8.320×10^-32^ | 2.588×10^-31^ | 2.273×10^-31^ |
| ***β_xzz_*** | -1.553×10^-31^ | -5.833×10^-31^ | -7.149×10^-31^ |
| ***β_yzz_*** | -6.378×10^-31^ | -6.489×10^-31^ | -5.055×10^-31^ |
| ***β_zzz_*** | -3.889×10^-32^ | -7.819×10^-31^ | -4.037×10^-31^ |
| ***β_total_*** | 5.565×10^-30^ | 5.240×10^-30^ | 6.317×10^-30^ |

**Table S9:** Percentage of fragments 1-3 for HOMOs and LUMOs of **A-C**.

| **Compounds** | **LUMO** | | | **HOMO** | | |
| --- | --- | --- | --- | --- | --- | --- |
|  | **Fragment 1** | **Fragment 2** | **Fragment 3** | **Fragment 1** | **Fragment 2** | **Fragment 3** |
| **A** | 53.5 | 40.8 | 5.7 | 46.8 | 51.5 | 1.7 |
| **B** | 59.9 | 39.4 | 0.7 | 36.1 | 60.8 | 3.1 |
| **C** | 0.0 | 0.4 | 99.6 | 49.7 | 49.6 | 0.7 |
